# Discovery of Demurilactone A: A Specific Growth Inhibitor of L-Form *Bacillus subtilis*

**DOI:** 10.1021/acsinfecdis.2c00220

**Published:** 2022-10-21

**Authors:** Yousef Dashti, Fatemeh Mazraati Tajabadi, Ling Juan Wu, Felaine Anne Sumang, Alexander Escasinas, Nicholas Edward Ellis Allenby, Jeff Errington

**Affiliations:** † The Centre for Bacterial Cell Biology, Biosciences Institute, Medical School, Newcastle University, Newcastle Upon Tyne NE2 4AX, U.K.; ‡ Odyssey Therapeutics Inc, The Biosphere, Draymans Way, Newcastle Helix, Newcastle Upon Tyne NE4 5BX, U.K.

**Keywords:** demurilactone A, polyketide synthases, Bacillus subtilis, L-form, insertional mutagenesis

## Abstract

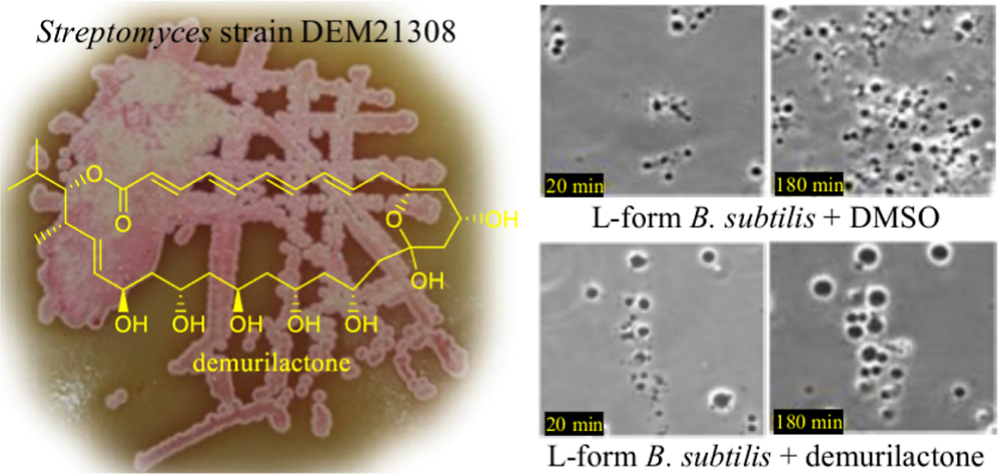

Metabolic profiling of the extracts from a library of actinobacteria led to the identification of a novel polyketide, demurilactone A, produced by *Streptomyces* strain DEM21308. The structure of the compound was assigned based on a detailed investigation of 1D/2D NMR spectra and HR-MS. Whole genome DNA sequencing, followed by bioinformatics analysis and insertional mutagenesis, identified type I polyketide synthases encoded by the *dml* gene cluster to direct the biosynthesis of this polyene macrolide. While the number of modules is consistent with the carbon backbone of the assigned structure, some discrepancies were identified in the domain organization of five modules. Close investigation of the amino acid sequences identified several mutations in the conserved motifs of nonfunctional domains. Furthermore, the absolute configuration of hydroxy-bearing stereocenters was proposed based on analyses of the ketoreductase domains. Remarkably, although demurilactone A has little detectable activity against normal-walled bacteria, it specifically inhibits the growth of cell wall-deficient “L-form” *Bacillus subtilis* at a minimum inhibitory concentration value of 16 μg/mL. Time-lapse microscopy analyses revealed that demurilactone affects membrane dynamics, probably by reducing membrane fluidity. This compound could be a powerful reagent for studying long-standing questions about the involvement of L-forms in recurrent infection.

## Introduction

Specialized metabolites possess remarkable structural and chemical diversities and have been a reservoir of life-saving therapeutics for various diseases.^1^ Modular polyketide synthases (PKS), nonribosomal peptide synthetases (NRPS), and their hybrids (PKS–NRPS) produce specialized metabolites with diverse structural features, which include some of the most important pharmaceuticals. Polyketide natural products made by type I modular PKS are the basis of about one-third of marketed medicines.^2^ While polyketides have remarkably diverse structures, the molecular logic for their biosynthesis is simple. Modules of type I PKSs successively elongate the polyketide chain, process, and finally terminate the biosynthesis.^3^ Each module consists of at least three domains, including acyltransferase (AT), an acyl carrier protein (ACP), and ketosynthase (KS) units, which collectively extend the polyketide chain by two carbon atoms. The AT domain loads the acyl units from acyl-coenzyme A (acyl-CoA) to the ACP, and KS domains are responsible for carbon–carbon bond formation between the acyl-ACP and the intermediate from the upstream module. In addition, PKS modules can also include ketoreductase (KR), dehydratase (DH), or enoyl reductase (ER) units, which successively reduce the β-keto group to a hydroxyl (KR), form a double bond via water removal (DH), and reduce the double bond to a single bond (ER). The final module contains a thioesterase (TE) that releases the polyketide chain through hydrolysis or cyclization.^3^

Peptidoglycan (PG) is the primary component of the bacterial cell wall, which is essential for the shape and structural integrity of the cell, and it creates a protective barrier against environmental factors.^4^ Inhibition of bacterial cell wall biosynthesis by either β-lactam antibiotics or immune factors such as lysozyme can induce switching of bacterial cells into the cell-wall-deficient or L-form state.^5,6^ L-forms are completely resistant to antibiotics targeting PG synthesis and are less susceptible to immune factors.^7,8^ Upon termination of treatment with antibiotics, they can switch back into the walled state, and it has been suggested that the ability of the pathogenic bacteria to switch to the L-form and revert to the wall state might be involved in the recurrence of infection.^9−13^ We have recently shown that L-form switching is a physiologically relevant phenomenon and possibly is responsible for recurrent urinary tract infections.^14^ Thus, to avoid the recurrence of infection, it is crucial to have antibiotics targeting L-form bacteria during bacterial treatment with wall-targeting antibiotics. Herein, the structure of a novel growth inhibitor of L-form *Bacillus subtilis*, which we named demurilactone A, is described, together with its proposed biosynthetic assembly by type I PKS.

## Results and Discussion

**Figure 1:**
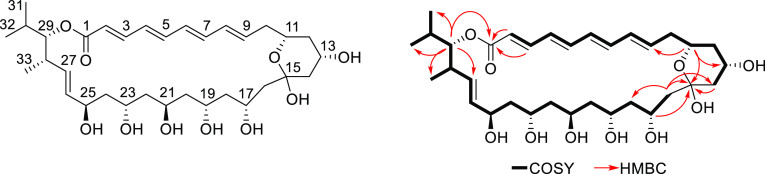
Structure of demurilactone A annotated with carbon numbers (left panel) and annotated with COSY and key HMBC correlations (right panel).

Metabolic profiling of an ethyl acetate extract of *Streptomyces* strain DEM21308 cultured on a solid GYM medium using positive ion ESI-Q-TOF-MS showed a peak corresponding to the molecular formulas of C_33_H_52_O_10_ (calculated for C_33_H_52_NaO_10_^+^: 631.3456, found: 631.3451). A mass-directed purification approach on a semipreparative HPLC-Q-MS was used to isolate the compound from an ethyl acetate extract obtained from a 1 L culture of the strain. Comparison of the obtained molecular formulas and ^1^H NMR signals of the compound with chemical databases suggested that this compound was potentially a novel molecule; therefore, we proceeded to assign the planar structure using 1D/2D NMR experiments (Figure 1; Tables S1 and S2, and Figures S3–S14).

The ^1^H NMR spectrum of the compound recorded in DMSO-*d*_6_ showed several overlapped proton signals. Shifting to a mixture of CD_3_OD/CDCl_3_ (3:1) helped resolve many of the peaks. Two spin systems were identified by correlation spectroscopy, HSQC, and HMBC correlations (Figure 1). HMBC correlations helped to link these fragments to each other. On the basis of observed ^2^*J* correlations of H14 (δ_H_ 1.98) and H16 (δ_H_ 1.65/1.73), as well as the ^3^*J* correlation of H17 (δ_H_ 4.19) to the quaternary carbon at δ_C_ 98.5, from one side, they are connected via C15. Similarly, the ^3^*J* correlation from H29 (δ_H_ 4.77), and the ^2^*J*/^3^*J* correlations of H2 (δ_H_ 5.83)/H3 (δ_H_ 7.26) to the carbonyl carbon at δ_C_ 169.3, established the existence of the ester bond that generates the 30-membered macrocycle ring. Based on its molecular formula, the compound has eight degrees of unsaturation, of which six are accounted for by five sets of olefins and one ester carbonyl. The remaining two degrees of unsaturation indicate the bicyclic nature of the molecule: one is the 30-membered macrocycle ring formed via the ester bond and the other is the hemiketal ring revealed by the ^3^*J* HMBC correlation of H11 (δ_H_ 4.02) to quaternary carbon C15 with the characteristic chemical shift at δ_C_ 98.5. All double bonds were assigned as *E*-configured, based on large coupling constants (*J* = ∼15) between the olefinic protons H2/H3, H4/H5, H6/H7, H8/H9, and H26/H27.

To identify the biosynthetic gene cluster (BGC) for demurilactone A, genomic DNA from the producer organism was isolated and sequenced using a combination of Nanopore and Illumina DNA sequencing methods. Bioinformatic analysis of assembled genome using antiSMASH^15^ revealed six putative PKS BGCs in the genome. Analysis of the module organization and domain specificities of these PKSs identified a putative BGC, annotated as *dml* (GenBank accession number ON123837), which contains five genes (*dmlE*, *dmlF*, *dmlG*, *dmlH,* and *dmlI*) encoding a total of 15 type I PKS modules (Table 1 and Figure 3). The *dml* gene cluster contains nine other genes encoding the following proteins: a thioesterase (*dmlJ*), four regulators (*dmlA*, *dmlC*, *dmlK*, and *dmlM*), a transporter (*dmlB*), a putative dihydrodipicolinate synthase (*dmlL*), an aminoacyl-tRNA editor (*dmlN*), and one hypothetical protein (*dmlD*). Further details are summarized in Table 1.

**Table 1:** Predicted Functions of the Proteins Encoded by the *dml* BGC

protein	Locus_tag	predicted function
DmlA	ctg4_73	transcriptional regulator-MarR family
DmlB	ctg4_74	transporter-EamA family
DmlC	ctg4_75	transcriptional regulator-MarR family
DmlD	ctg4_76	hypothetical protein
DmlE	ctg4_77	PKS
DmlF	ctg4_78	PKS
DmlG	ctg4_79	PKS
DmlH	ctg4_80	PKS
DmlI	ctg4_81	PKS
DmlJ	ctg4_82	thioesterase
DmlK	ctg4_83	LuxR family DNA-binding response regulator
DmlL	ctg4_84	dihydrodipicolinate synthase family
DmlM	ctg4_85	LysR family transcriptional regulator
DmlN	ctg4_86	aminoacyl-tRNA editing domain

**Figure 2:**
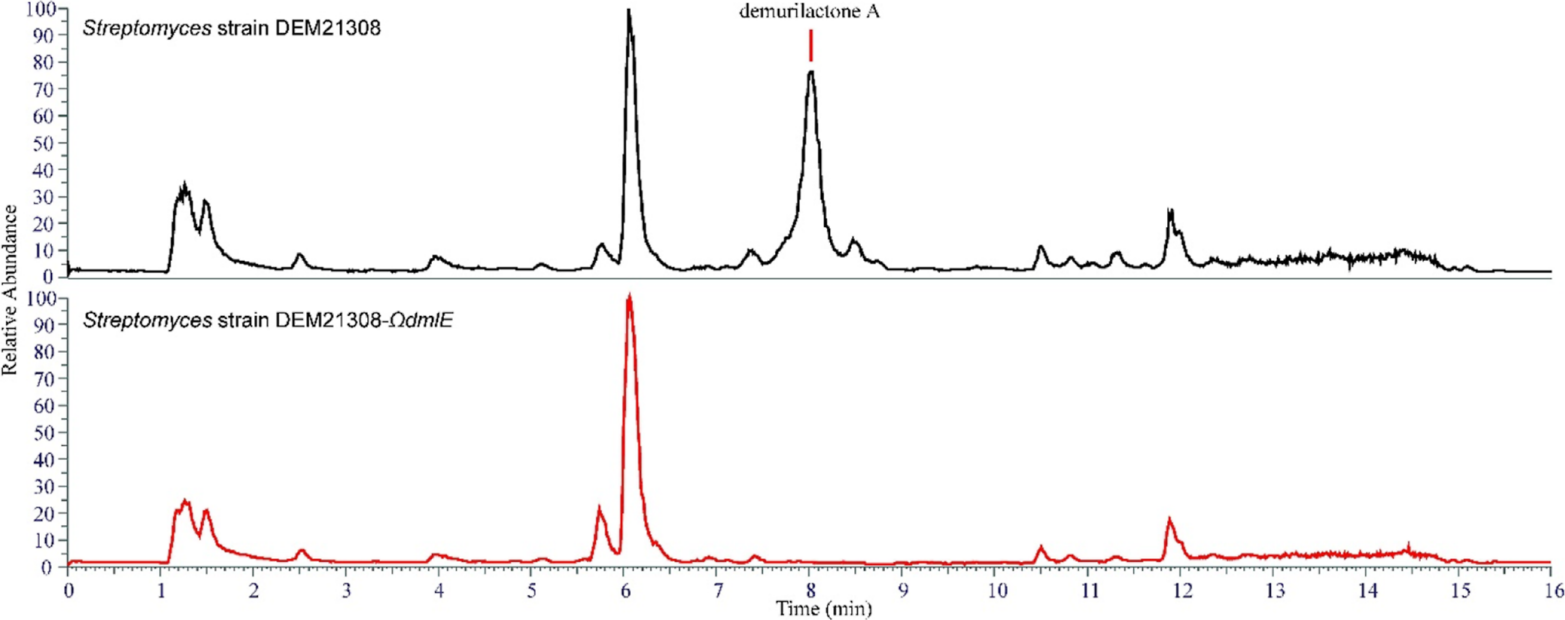
Comparison of LC–MS profile of culture extracts from *Streptomyces* strain DEM21308 and *Streptomyces* strain DEM21308-Ω*dmlE*. Insertional mutagenesis in *dmlE* verified the involvement of the *dml* gene cluster in demurilactone A biosynthesis.

To confirm the involvement of the *dml* gene cluster in demurilactone A production, we inactivated *dmlE* via insertional mutagenesis. For this purpose, we created a new vector based on pSET152, in which phage integrase (ΦC31) and attP sites were replaced with a target nucleotide sequence from within *dmlE*. Liquid chromatography–mass spectrometry (LC–MS) analysis confirmed that the production of demurilactone A was abolished in the mutant strain (Figure 2).

**Figure 3:**
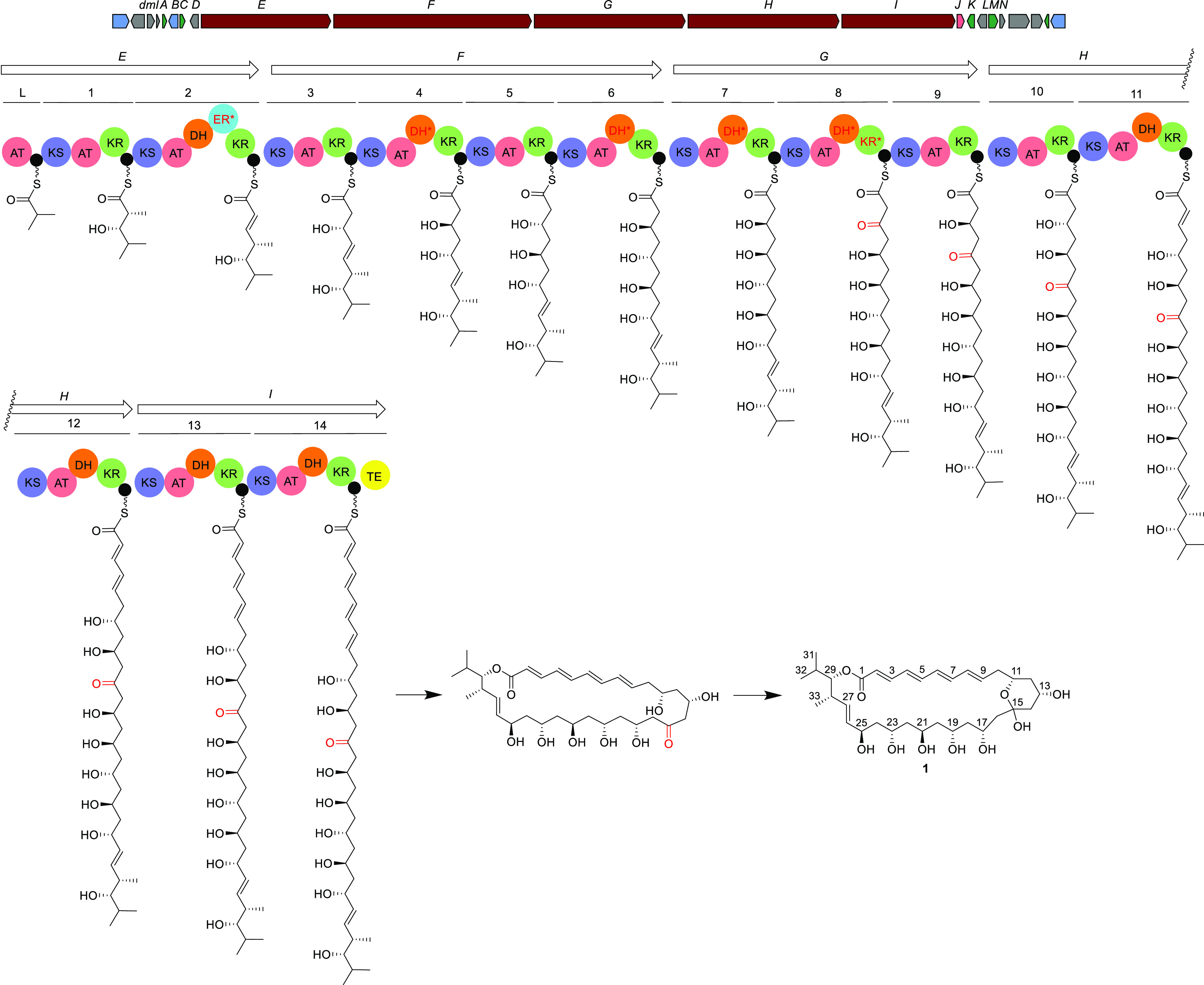
*dml* gene cluster and proposed biosynthetic pathway of demurilactone A. Abbreviations: AT; acyltransferase, KS; ketosynthase, KR; ketoreductase, DH; dehydratase, ER; enoyl reductase, TE; thioesterase. ACPs are shown in black circles. Inactive domains are shown with an asterisk in red.

The five modular PKS enzymes, DmlE–DmlI, are predicted to provide a loading module and 14 extender modules, which would generate the 30-carbon polyketide backbone via incorporation of 1 methyl malonyl-CoA and 13 malonyl-CoA extender units onto a priming isobutyl unit. This would be cyclized and released from the assembly line, catalyzed by the TE domain at the C-terminus of module 14 (Figure 3). Multiple sequence alignments were used to predict the substrate specificity of the AT domains.^16,17^ With the exception of the loading and AT modules one and six, all modules showed a His-Ala-Phe-His motif that is specific for malonyl-CoA,^17^ consistent with the assigned structure (Table S3). Module one is predicted to be specific for methyl malonyl-CoA due to the presence of a Tyr-Ala-Ser-His motif. The AT of module six has a Tyr-Ala-Pro-His motif which, based on the structure of demurilactone A, should be specific for malonyl-CoA. A comparison of the structure with the domain organization of the *dml* BGC suggested that the ER of module two and the DH of modules four, six, seven, and eight should be inactive (Figure 3).

Multiple sequence alignments of the ER and several active ERs from well-characterized biosynthetic systems revealed a shorted sequence and multiple mutations in the NADPH binding motif. Thus, the conserved HAAAGGVGMA motif of functional ERs appears as HAPTGDVGAA in the DmlE ER protein (Table S4). It seems likely that these amino acid substitutions in the NADPH binding motif of the ER would disrupt the binding cleft and therefore impede accommodation of NADPH, and consequently make the ER nonfunctional. Donadio et al.^18^ previously showed that mutagenesis of the coding region for the NADPH binding site in an ER could be used to generate an erythromycin analogue.

DH domains of PKS systems are recognizable by their signature HXXXGXXXXP motif near their N-terminus in which catalytic histidine is invariant.^19^ Multiple sequence alignments of all active and inactive DH domains of *dml* BGC revealed that the histidine in the HXXXGXXXXP motif is replaced with tyrosine in DH modules six and eight (Table S5), which explains the nonfunctional nature of these two domains. DH modules four and seven both had substitutions in the conserved GYXYGPXF motif, in which the first glycine in the sequence is replaced with aspartic acid. In addition, module seven showed the replacement of phenylalanine in the expected LPFXW motif with valine. It has been hypothesized that this phenylalanine makes contact with the ACP.^19^ Further experiments would be required to test whether these substitutions are sufficient to inactivate DH modules four and seven, but the deduced structure of demurilactone A strongly suggests that this is the case.

Subsequently, multiple sequence alignments of the KR domains were used to predict the absolute configuration of the hydroxy-bearing stereocenters of demurilactone A, based on the conserved regions of KRs that direct the stereochemistry.^20−24^ This approach has been used to propose the absolute configuration of several macrolides including termidomycin A,^25^ niphimycins,^26^ brasilinolides,^27^ caniferolides,^28^ and quinolidomicin.^29^ Based on the presence of the Leu-Asp-Asp (LDD) motif in the loop region and the absence of tryptophan (W) and the YXP motifs in the catalytic region, the KRs of modules 4, 6, 7, 9, 11, 12, 13, and 14 were assigned as B1 type (Table S6). Therefore, the chiral centers C-23, C-19, C-17, and C-13 are assigned as “*S*”. Furthermore, the B1 type assignment of modules 11–14 is consistent with *trans*-double bounds C-2/C-3, C-4/C-5, C-6/C-7, and C-8/C-9, as *cis* configured double bonds are only generated by a DH that operates on the product of an A-type KR.^21^ Similarly, the KR domains of modules 1, 3, 5, and 10 were assigned as A1 type based on the absence of an LDD motif in the loop region and the presence of tryptophan (W) and lack of histidine (H), in the catalytic region (Table S6). This led us to propose the stereochemistry on C-29, C-25, C-21, and C-11 as “*R*”. Generation of the six-membered hemiketal ring of demurilactone A requires a carbonyl group on C15; therefore, the KR domain of module 8 must be inactive or skipped during the biosynthetic assembly of the core polyketide (Figure 3). Investigation of the active site of the KR of module 8, however, indicated that this domain harbors a functional active site. The process leading to the intramolecular cyclization for hemiketal formation remains to be investigated.

The similarity of demurilactone A to polyketide antifungal agents such as nystatin prompted us to test for possible antibiotic activity. The compound was tested against *Candida albicans*, *Schizosaccharomyces pombe*, *Saccharomyces cerevisiae*, methicillin-resistant *Staphylococcus aureus*, and *B. subtilis*, but no growth inhibitory effects were detected. Serendipitously, and unexpectedly, we found that the compound was highly active against L-form *B. subtilis*. Figure S16 shows a clear zone around the disc containing demurilactone A on a lawn of L-form *B. subtilis*. When tested in a liquid medium, demurilactone A had a minimum inhibitory concentration value of 16 μg/mL for L-form *B. subtilis*, while normal (walled) *B. subtilis* showed no growth inhibition even at 100 μg/mL, confirming that demurilactone A was differentially active against L-forms (Figure S17). Prolonged incubation of demurilactone A with L-forms did result in some growth at concentrations 16 and 20 μg/mL but not at 30 and 40 μg/mL, suggesting that the compound is bactericidal at higher concentrations (Figure S17).

**Figure 4:**
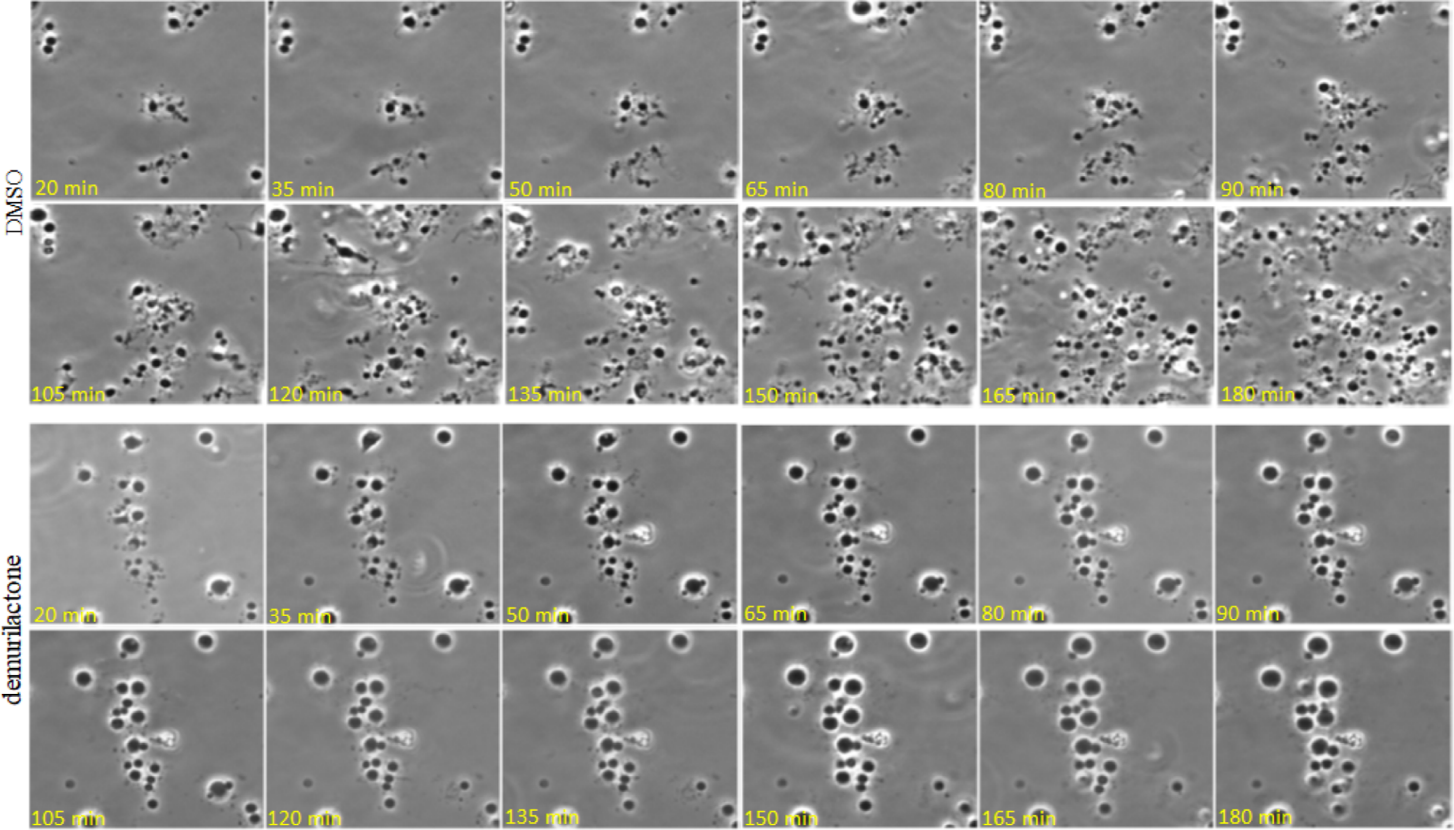
Inhibitory effect of demurilactone A on L-form *B. subtilis* proliferation (bottom panel) compared to the DMSO control (top panel). The first images shown were taken 20 min after the addition of DMSO (1 μL in 100 μL of cells) or demurilactone A (2 μL of demurilactone A at 1.6 μg/mL (diluted in NB/MSM) in 100 μL of cells), and the last images were taken at 180 min.

To understand the mode of action of demurilactone A, we carried out time-lapse microscopy to observe the effects of the compound on L-form growth. Figure 4 shows the growth of L-form *B. subtilis* in the presence of DMSO (as a control) or demurilactone A. As expected, DMSO had no effect on growth. L-form cells continued to grow and divide after the addition of DMSO, and by 180 min, the field was already filled with cells. In the presence of demurilactone A, however, cells continued to grow larger but did not divide, such that 180 min after the addition of the compound, the cells had substantially enlarged without much of an increase in cell number. Division of L-form bacteria is independent of the FtsZ-based cell division machinery that is essential for walled bacteria.^7,8,13,30^ Instead, they divide by a variety of processes involving membrane blebbing, tubulation, vesiculation, and fission, which is driven by excess membrane synthesis and is sensitive to membrane fluidity.^8,31^ As the cells were still able to grow larger in the presence of demurilactone A, we assumed that excess membrane synthesis continued and that the inhibitory effect was mainly due to the loss of the dynamic biophysical properties of the membranes. Indeed, examination of cells very soon after the addition of demurilactone A revealed that within 2 min of the addition of the compound, cells started to become round and lost the dynamic shape changes that normally associate with actively dividing L-form cells (Figure S18). Taken together, these analyses suggest that demurilactone A inhibits the growth of L-form bacteria by reducing membrane dynamics, possibly by reducing membrane fluidity. Previous studies have shown that L-form *B. subtilis* cells are hypersensitive to peptide antibiotics nisin and daptomycin;^32^ however, these two compounds also have lethal effects on walled *B. subtilis*. To our knowledge, this is the first natural product identified to inhibit the growth of L-form but not walled *B. subtilis*. Interestingly, the closely related polyketide antifungal antibiotic, nystatin, did not show growth inhibitory activity against L-form *B. subtilis*.

## Conclusions

Evidence is growing for L-form bacteria as a source of antibiotic resistance, especially in recurrent infections.^9−14^ Tackling this problem requires the use of combination chemotherapy including antibiotics targeting bacteria in the L-form state. The novel macrolide demurilactone A identified from a culture extract of *Streptomyces* DEM21308 is a potential starting point for novel drugs acting on L-form-dependent recurrent infection. Bioinformatics analysis of the sequenced genome of *Streptomyces* DEM21308 identified the putative *dml* BGC responsible for the assembly of demurilactone A, which was verified by insertional mutagenesis. Detailed investigation of the multiple sequence alignments revealed several inactive domains consistent with the structure assigned by HR-MS and NMR analysis.

## Supporting Information Available

General experimental procedures; procedures for compound production and purification, genomic DNA sequencing and assembly, insertional mutagenesis, and antibacterial assays; NMR data and spectra; and multiple sequence alignments (PDF)
