# Supplementary material for: Discovery of Demurilactone A: A Specific Growth Inhibitor of L-Form Bacillus subtilis
Source: ACS Infect Dis. 2022 Oct 21;8(11):2253–8. doi: 10.1021/acsinfecdis.2c00220 (PMC9673147; doi:10.1021/acsinfecdis.2c00220)
Supplement: Supplementary file 1 — id2c00220_si_001.pdf [file id2c00220_si_001.pdf]

Discovery of demurilactone A; a specific growth inhibitor of L-form *Bacillus subtilis*

Yousef Dashti[1,3,*], Fatemeh Mazraati Tajabadi[2], Ling Juan Wu[1], Felaine Anne Sumang[1], Alexander Escasinas[2], Nicholas Edward Ellis Allenby[2], Jeff Errington[1,2,3,*]

[1] The Centre for Bacterial Cell Biology, Biosciences Institute, Medical School, Newcastle University, Newcastle upon Tyne, NE2 4AX, UK
[2] Odyssey Therapeutics Inc., The Biosphere, Draymans Way, Newcastle Helix, Newcastle upon Tyne, NE4 5BX, UK
[3] Present address, Biomedical Building C81, Faculty of Medicine and Health, University of Sydney, Sydney NSW 2015, Australia
[*]Corresponding authors: yousef.dashti@sydney.edu.au, dashti.yousef@gmail.com and jeffery.errington@sydney.edu.au

**Contents**	**Page**
Materials and methods	S2-S7
Figure S1. Structures of demurilactone A with numbering used in NMR tables S1 and S2.	S8
Table S1. ^{1}H and ^{13}C NMR chemical shifts, and HMBC correlations of demurilactone A in $CDCl_3$/CD_3OD (3:1).	S8
Table S2. ^{1}H and ^{13}C NMR chemical shifts of demurilactone A in DMSO-d_6.	S9
Table S3. Sequences of primers used to construct pSET-*dmlE* vector.	S10
Table S4. Sequences of primers used to validate the insertion site within the genome of *Streptomyces* strain 21308.	S10
Figure S2. Agarose gel of the PCR products that verified the position of inserted vector in the genome of *Streptomyces* strain 21308 Ω*dmlE*.	S10
Table S5. Multiple sequence alignment of AT domains	S11-S12
Table S6. Multiple sequence alignment of ER domains	S12
Table S7. Multiple sequence alignment of DH domains	S13
Table S8. Multiple sequence alignment of KR domains	S14
Figures S3-S8. 1D/2D spectra of demurilactone A in CD_3OD/$CDCl_3$ (3:1).	S15-S20
Figures S9-S14. 1D/2D spectra of demurilactone A in DMSO-d_6.	S21-S26
Figure S15. HR-MS spectrum of demurilactone A.	S27
Figure S16. Comparative growth inhibitory experiments of penicillin G, rifampicin, and demurilactone A against L-form and walled *B. subtilis*.	S28
Table S9. Zone of inhibition of three replicates of penecilium G, rifampicin, demurilactone A, and DMSO against walled and L-form *Bacillus subtilis*.	S28
Figure S17. Growth curves of the L-form and walled *B. subtilis* in the presence of demurilactone A.	S29
Figure S18. Time-lapse images of L-form *B. subtilis* before and soon after adding demurilactone A	S30

Materials and Methods

General Experimental Procedures: NMR spectra were recorded on a Bruker Avance III 700 MHz spectrometer equipped with a TCI cryoprobe at 25 °C. The ^{1}H and ^{13}C NMR chemical shifts were referenced to the solvent peaks CD_3OD at δ_H 3.31 and δ_C 49.00 or DMSO-d_6 at δ_H 2.50 and δ_C 39.51. LC-ESI-Q-TOF-MS analyses were performed on an Agilent 1200 series connected to the C18 column Raptor ARC-18 (150 × 2.1 mm, 2.7 μm), coupled to a Bruker microTOF II mass spectrometer. Mobile phases consisted of water (0.1% formic acid) and acetonitrile (0.1% formic acid). The LC method was started with isocratic conditions of 5% acetonitrile for 5 min, followed by a gradient run of 5 to100% acetonitrile over 50 minutes, and finished by an isocratic flow for 5 minutes at a flow rate of 0.2 mL/min. The mass spectrometer was operated in positive ion mode with a scan range of 50-2500 *m/z*. IR spectrum recorded on a PerkinElmer UATR Two FT-IR Spectrometer. PoLAAr 2001 polarimeter was used for measuring optical rotation.

Production, Extraction and HPLC Purification of demurilactone A: *Streptomyces* strain DEM21308 was grown in 1 L liquid GYM medium consisting of 4 g/L glucose, 4 g/L yeast extract, 10 g/L malt extract, for 7 days at 30 °C. Cells were removed by centrifugation followed by filtration on a filter paper. Metabolites were extracted with ethyl acetate twice, combined and dried on a rotary evaporator. Dried extract was resuspended in 1 mL methanol, pre-adsorbed on dental cotton and dried by nitrogen gas. The dental cotton containing extract was then loaded into an HPLC guard cartridge (10 × 30 mm) attached to a semi-preparative reverse-phase C18 Betasil column (21.2 mm × 150 mm). Compound purification was performed on an Agilent 1260 Infinity II preparative HPLC connected to a single-Q mass spectrometer using the following HPLC method: initial isocratic conditions of 5% acetonitrile for 5 min, followed by a linear gradient from 5 to 100% acetonitrile over 45 min; then, continued by isocratic flow

for an additional 10 min at a flow rate of 12 mL/min. The fraction collector was set to collect fractions in 30 sec increments over 60 min run time. Demurilactone A was eluted in fractions 60 and 61. Purity of demurilactone A was over 95% determined by ^{1}H NMR.

demurilactone A: white solid (12 mg/L); ^{1}H NMR (700 MHz, $CD_3OD/CDCl_3$ (3:1)) and ^{13}C NMR (175 MHz, $CD_3OD/CDCl_3$ (3:1)), see Table S1; ^{1}H NMR (700 MHz, DMSO-d_6) and ^{13}C NMR (175 MHz, DMSO-d_6), see Table S2; $[\alpha]^{22}_{D}$ -82 (c 0.195 mg/mL, MeOH); IR(neat): $\upsilon max/cm^{-1}$ 3363.6, 2939.3, 1681.4, 1596.1, 1370.8, 1327.4, 1257.7, 1128.8, 1007.9; HRESIMS *m/z* 631.3451 $[M + Na]^+$ (calcd for $C_{33}H_{52}NaO_{10}^+$, 631.3456).

Genomic DNA Extraction, Genome Sequencing, Assembly, Error Correction, and Annotation: *Streptomyces* strain DEM21308 was inoculated in 10 mL liquid GYM culture medium and incubated for 3 days at 30 °C in 120 rpm. 300 µL of the cell culture was used for genomic DNA extraction using the Quick-DNA HMW MagBead Kit (Zymo Research, cat. no. D6060). DNA quality was assessed by agarose gel electrophoresis to ensure the absence of RNA. DNA concentration was assessed using the dsDNA assay on a Qubit fluorometer. *Streptomyces* strain DEM21308 was a part of a multiplexed nanopore MinION sequencing run (12 strains in total) using the Native Barcoding Expansion 1-12 (EXP-NDB104) and Ligation Sequencing Kit (SQK-LSK109). DNA library preparation, barcoding, and adapter ligation was performed according to the manufacturer's instructions. Illumina sequencing was performed at MicrobesNG, The BioHub, Birmingham.

The MinION long reads were assembled using Flye (version 2.8.1)[1] and error-corrected using minimap2 (version 2.17),[2] Racon (version 1.4.13)[3] and medaka (version 1.0.3).[4] Further error correction and polishing was accomplished by aligning Illumina MiSeq short reads to the Flye assembly using BWA-MEM (version 0.7.17)[5] and subsequently polished using Pilon (version 1.24).[6]

Inactivation of *dml* gene cluster via insertional mutagenesis: Single-crossover insertional mutagenesis was used to disrupt the production of demurilactone A in *Streptomyces* strain 21308. A 5.5 kbp vector was constructed by assembling a 2 kbp fragment from within *dmlE* and a 3.5 kbp fragment from pSET152 containing the *E. coli* origin of replication, apramycin resistance gene, and the traJ gene for conjugative transfer into the target strain. Sequences of the primer pairs used to amplify both fragments are shown in table S3. The two pieces were assembled using NEBuilder following manufacturer's protocol and the assembled product was transformed to *E. coli* DH10β and spread on LB plates containing 50 mg/mL apramycin. To validate the integrity of the construct (named pSET-*dmlE*), a few colonies were picked for plasmid extraction and sequencing. The validated vector was then transformed into ET12567[pUZ8002] for conjugation into the *Streptomyces* strain 21308 using the following standard procedure. The *E. coli* strain ET12567[pUZ8002] containing pSET-*dmlE* was grown overnight in 10 mL LB with kanamycin (50 mg/ml), chloramphenicol (35 mg/ml) and apramycin (50 mg/ml). The culture was refreshed with the same antibiotic combination and grown to OD_{600} 0.4. The culture was then washed twice with LB without antibiotics; the cell pellet was resuspended with 100 µL LB, mixed with *Streptomyces* strain 21308 spores, which had been pre-germinated by incubation at 50 °C for 15 minutes. The mixture was spread on Soy flour Mannitol (SFM) medium (soy flour 20 g/L, mannitol 20 g/L, agar 20 g/L) containing 10 mM $MgCl_2$ and incubated overnight at 30 °C before overlaying with nalidixic acid (25 mg/ml) and apramycin (30 mg/ml) and incubated again at 30 °C for 2-3 days. To validate the position of the single crossover homologous insertion in the genome of *Streptomyces* strain 21308, ten colonies were picked and patched on SFM plates containing nalidixic acid (25 mg/ml) and apramycin (30 mg/ml) for PCR and demurilactone production checks. The pair of primers on table S4 were used to identify the target mutant and the resulting amplified bands (Figure S2) were sent for sequencing to confirm insertion site. In addition, mutants along with

wild type *Streptomyces* strain 21308, were grown on solid GYM media for five days and demurilactone production was check by UHPLC-MS. The generated non-demurilactone producing mutant was named *Streptomyces* strain 21308 Ω*dmlE.*

Generation and growth of L-form *B. subtilis*: *B. subtilis* L-forms were grown in osmoprotective liquid medium NB/MSM or on NB/MSM agar plates. NB/MSM medium was composed of 2x magnesium-sucrose-maleic acid (MSM) pH7 (40 mM $MgCl_2$, 1 M sucrose, and 40 mM maleic acid) mixed 1:1 with 2 x nutrient broth (NB). *B. subtilis* L-forms were generated from strain RM121 (168CA *Δ18::tet* pLOSS-P_{spac}-*murC erm*).[7-8]

Disc diffusion assay for compound sensitivity: Exponentially growing *B. subtilis* L-form culture (strain RM121) and walled wild type culture (168ca) were diluted to an OD_{600} of 0.02 in the same medium (NB/MSM). Then 100 µl of the diluted walled cell culture or 200 µl diluted L-form culture were mixed gently with 10 ml molten 'soft' agar (NB/MSM/0.375% nutrient agar) before being poured onto solidified MSM/nutrient agar (50 ml; 1:1 mix of 2x MSM and 2x nutrient agar). When the cell-containing top layer had set, sterile filter paper discs containing penicillin G (50 µg), rifampicin (0.5 µg), nystatin (64 µg), demurilactone A (64 µg), and DMSO (5 µl) were put on the agar, and the plates were incubated at 30 °C for 1 day for walled cells and 3 days for L-forms.

Determination of MIC and growth curve using 96-well microtitre plates: An exponentially growing culture of the wild type strain 168ca and an overnight L-form culture, both grown in NB/MSM, were diluted in the same medium, and grown until the cultures reached exponential phase. Then the culture of the walled cells (168ca) was diluted into NB/MSM to an OD_{600} of 0.001, and the L-form culture to an OD_{600} of 0.002. Next, 100 µl of each diluted culture was added to 100 µl of NB/MSM containing different concentrations of the compound or DMSO (as a control) in each well. The final concentrations tested for L-forms were 0.25, 0.5, 2, 4, 8,

12, 16, 20, and 30 µl/ml; and 4, 8, 12, 16, 20, 30, 40, 60, 80, 90, 100 µg/ml for the wild type strain 168ca. The final concentration of DMSO in each well was 0.47%. The OD_{600} of each well was read every 320 seconds in a BMG Fluostar Optima plate reader set at 37 °C. Samples were triplicated.

Microscopy: Time-lapse microscopy was carried out using CELLview™ Culture dish (35 mm) from Greiner Bio-One Ltd. Exponentially growing L-form culture was diluted (1 in 5) in fresh NB/MSM medium, then 100 µl was added into each of the compartments of the dish. The dish loaded with cells was incubated for about 30 min at 30 °C to allow the cells to settle and stick to the glass bottom. Then 50 ul of the culture in the compartment was removed and replaced with 50 µl fresh NB/MSM medium to reduce the number of 'floating' cells. Time-lapse imaging could now start. Phase-contrast images were captured from several positions in each compartment every 3 min for about 30 min to ensure that cells were growing and dividing. Then, time-lapse imaging was paused and, with the dish remained on the microscope stage, compounds were carefully added into the cells, and imaging was continued. With care, cells would remain in position and in focus, so images of the cells both before and after adding the compounds were captured. The microscope used was a Nikon Eclipse Ti equipped with CoolLed pE-4000 LED light source and a Photometrics BSI sCMOS camera, and a Nikon Plan Apo 100×/1.40 NA Oil Ph3 objective. Temperature control was set at 32 °C. 2 µl of Demurilactone A at 1.6 µg/ml (diluted in NB/MSM) was added into 100 µl cells; for the control, 1 µl DMSO was added to 100 µl of cells.

References:

1. Kolmogorov, K.; Yuan, J.; Lin, Y.; Pevzner, P. Assembly of long error-prone reads using repeat graphs. *Nat. Biotechnol*. **2019**, *37*, 540-546.
2. Li, H. Minimap2: pairwise alignment for nucleotide sequences. *Bioinformatics* **2018**, *34*, 3094-3100.
3. Vaser, R.; Sović, I.; Nagarajan, N.; Šikić, M. Fast and accurate de novo genome assembly from long uncorrected reads. *Genome Res*. **2017**, *27(5)*, 737-746.
4. Medaka, https://github.com/nanoporetech/medaka; Oxford Nanopore Technologies Ltd.

5. Li, H. Aligning sequence reads, clone sequences and assembly contigs with BWA-MEM. **2013**, arXiv:1303.3997v2 [q-bio.GN].
6. Walker, B.J.; Abeel, T.; Shea, T.; Priest, M.; Abouelliel, A.; Sakthikumar, S.; Cuomo, C.A.; Zeng, Q.; Wortman, J.; Young, S.K.; Earl, A.M. Pilon: An integrated tool for comprehensive microbial variant detection and genome assembly improvement. *PLoS ONE* **2014**, *9(11)*: e112963.
7. Mercier, R.; Kawai, Y.; Errington, J. Excess membrane synthesis drives a primitive mode of cell proliferation. *Cell* **2013**, *152(5)*, 997-1007.
8. Wu, L.J.; Lee, S.; Park, S.; Eland, L.E.; Wipat, A.; Holden, S.; Errington, J. Geometric principles underlying the proliferation of a model cell system. *Nat. Commun.* **2020**, 11(1):4149.

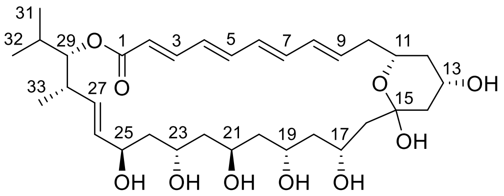

Figure S1. Structure of demurilactone A with numbering used in NMR tables S1 and S2.

Table S1.$^{1}H/^{13}C$ NMR chemical shifts and HMBC correlations of demurilactone A in $CD_3OD/CDCl_3$ (3:1).

Position	δ_C (175 MHz)	δ_H (700 MHz), mult (*J* in Hz)	HMBC connection
1	169.3		
2	120.9	5.83, d (15.1)	C1, C4
3	146.8	7.26, dd (15.1, 11.4)	C1, C2, C4, C5
4	129.9	6.34, dd (14.8, 11.4)	C2, C3, C6
5	142.9	6.63, dd (14.8, 11.0)	C3, C6, C7
6	130.7	6.25, dd (14.8, 11.0)	C4, C5, C8
7	138.9	6.43, dd (14.8, 10.8)	C5, C8, C9
8	132.8	6.19, ddd (14.9, 10.8, 1.8)	C6, C7, C10
9	136.5	5.82, m	C7, C10, C11
10	40.8	2.22, dt (15.1, 10.3)<br>2.40, brd (4.7)	C8, C9, C11, C12
11	68. 5	4.02, m	C9, C10, C13, C15
12	41.9	1.11, m<br>1.93, m	C10, C11, C13, C14
13	64.7	4.06, m	
14	45.7	1.21, m<br>1.98, ddd (12.2, 4.7, 1.7)	C12, C13, C15
15	98.5		
16	48.8	1.65, dd (14.2, 4.3)<br>1.73, dd (14.2, 8.2)	C14, C15, C17, C18
17	67.2	4.19, m	C15, C16, C18, C19
18	46.4	1.57, m	C16, C17, C19, C20
19	67.1	3.96, m	C17, C21
20	44.6	1.18, m<br>1.46, ddd (14.2, 10.4, 2.0)	C18, C21
21	65.3	4.14, m	C19, C20, C22, C23
22	46.9	1.19, m<br>1.29, ddd (14.3, 10.5, 2.3)	C20, C21, C24
23	65.5	3.98, m	C21, C24, C25
24	46.3	1.16, m<br>1.37, ddd (14.5, 11.1, 2.3)	C22, C23, C26
25	68.6	4.34, m	C23, C24, C26, C27
26	134.3	5.39, ddd (15.7, 3.6, 1.7)	C24, C25, C28
27	130.7	5.69, ddd (15.8, 5.0, 1.9)	C25, C28, C29, C33
28	37.5	2.63, m	C26, C27, C33
29	81.6	4.77, dd (9.7, 2.4)	C1, C27, C28, C30, C31, C32, C33
30	30.2	1.93, m	C29, C31, C32
31	19.2	0.97, d (*J* = 6.7)	C29, C30, C32
32	20.3	0.90, d (*J* = 6.6)	C29, C30, C31
33	10.5	1.04, d (*J* = 6.9)	C27, C29, C32

Table S2.^1H and ^{13}C NMR chemical shifts of demurilactone A in DMSO-d_6.

Position	δ_C (175 MHz)	δ_H (700 MHz), mult (J in Hz)
1	166.2	
2	120.2	5.84, d (15.2)
3	144.7	7.14, dd (15.1, 11.4)
4	128.9	6.36, dd (14.7, 11.5)
5	141.2	6.65, dd (14.8, 11.2)
6	129.5	6. 24, dd (14.7, 11.2)
7	137.6	6.42 dd (14.8, 10.9)
8	131.1	6.17, dd (15.0, 11.0)
9	136.23	5.85, m
10	40.0	2.13, m<br>2.32, m
11	67.1	3.84, m
12	41.4	0.95, *ol*<br>1.78, *ol*
13	62.6	3.86, *ol*
14	45.7	1.07, *ol*<br>1.78, *ol*
15	96.8	
16	49.3	1.52, dd (14.0, 6.1)<br>1.60, *ol*
17	66.2	4.03, m
18	45.4	1.32, m<br>1.59, *ol*
19	65.9	3.87, *ol*
20	45.0	0.99, *ol*<br>1.24, ddd (12.7, 10.3, 2.0)
21	62.8	3.39, m
22	46.9	0.98, *ol*<br>1.05, *ol*
23	63.1	3.80, m
24	46.4	0.94, m<br>1.10, m
25	66.1	4.19, d (10.5)
26	134.7	5.28, dd (15.5, 3.6)
27	128.3	5.55, dd (15.6, 5.1)
28	36.0	2.52, m
29	79.3	4.66, d (9.46)
30	28.7	1.85, m
31	18.9	0.92, d, (6.5)
32	19.8	0.83, d, (6.5)
33	10.9	0.97, d, (6.8)

Table S3. Sequences of primers used for construction of pSET-*dmlE* vector.

Primer Name	Sequences
pSET-*dmlE*-F	CTGGTACCAGAACCTGCGCACGAGACACCCGGGAAGCCTG
pSET-*dmlE*-R	AGCGCTTCCCAGGAGATCTCAGCTTGCATGCCTGCAGGTC
dmlE-pSET-F	GACCTGCAGGCATGCAAGCTGAGATCTCCTGGGAAGCGCT
dmlE-pSET-R	CAGGCTTCCCGGGTGTCTCGTGCGCAGGTTCTGGTACCAG

Table S4. Sequences of primers used to validate the insertion site within the genome of *Streptomyces* strain 21308.

Primer Name	Sequences
check1-dmlE-F	GACCTGGACGGGCGCTAC
check1-dmlE-R	CTCTAGAGTCGACCTGCAGC
check2-dmlE-F	GCCAGGTGCGAATAAGGGAC
check2-dmlE-R	GCCGTCTCCTCCAGCCAGAA

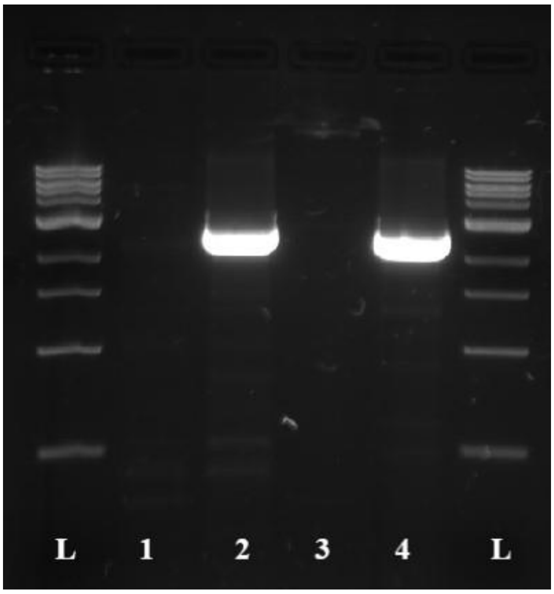

Figure S2. Agarose gel of PCR products that verified the position of inserted vector in the genome of *Streptomyces* strain 21308-Ω*dmlE*. **L:** Ladder; **1:** *Streptomyces* strain 21308 genome with primer pair check1 (negative control); **2:** *Streptomyces* strain 21308-Ω*dmlE* genome with primer pairs check1; **3:** *Streptomyces* strain 21308 genome with primer pair check2 (negative control); **4:** *Streptomyces* strain 21308-Ω*dmlE* genome with primer pairs check2.

Table S5. Multiple sequence alignment of AT domains from *dml* biosynthetic gene cluster.

```
AT_M13          LFSGQGSQRVGMGRELYGEFPVFGQALEEVCAALDPHLERPLRDVLFAEPGTEAAALLDL    60
AT_M2           LFSGQGSQRIGMGRGLYAAHPVFADALDEVFAHLDPLLALPLRDVVFGEDQ----ALLDR    56
AT_M9           LFSGQGSQRLGMGRELADRFPVFAESLDRVLAEFGPE----VREVLLGDDAD--ADALNQ    54
AT_M3           LFSGQGSQRLGMGRELADRFPVFADALDEVLAEFGPE----VREVLFGEDA----DALNE    52
AT_M7           LFSGQGSQRLGMGRELASRFPVFADALDETLGEFDPG----VREVLFGEDA----DALNE    52
AT_M10          LFSGQGSQRLGMGRELADRFPVFADALDEVLAEFDPR----VREILRGDDA----DALNE    52
AT_M11          LFSGQGSQRLGMGRELADRFPVFAEALDEVLAEFDPG----VREALFGDDA----DVLNQ    52
AT_M8           LFSGQGSQRLGMGRELADRFPVFAEALDRVLAEFGPE----VREVLFGEDA----DALNE    52
AT_M14          LFSGQGSQRLGMGRELADRFPVFAEALDRVLAEFGPE----VREVLFGEDA----DALNE    52
AT_M4           LFSGQGSQRLGMGRELADRFPVFAEALDEVLAEFGPE----VREVLFGEDA----DALNE    52
AT_M5           LFSGQGSQRLGMGRELADHFPVFAEALDRVLAEFGPE----VRDVLFGEDA----DALNE    52
AT_M12          LFSGQGSQRLGMGRELADRFPVFAEALDQVLAEFGPV----VRDVLFGEDA----DALNE    52
AT_L            VFPGHGPQWLGMADELLAASPVFAENLHACAEALTPYVDWPVLDVLRGRTDT---PSLDR    57
AT_M1           VFPGQGSQWLGMGAALAESSPVFRASLDACAEALAPHVDWSLHDVLND---A---AALER    54
AT_M6           VFPGHGSQWVGMALELLESSPVFRARMQECARALEEHVDWTLLAAIRD---P---ELMER    54
                :* *:* * :**.  *    ***   :.     :       :   :           ::
AT_M13          TTYTQPALFAIEVALFRLVTAWGLAPHYLAGHSIGELAAAHVSGVFSLADACALVAARGR    120
AT_M2           TEYAQPALFAVEVALYRLWTSWGLRPDALAGHSIGELAAAHVAGVFSLADACAVVAARGA    116
AT_M9           TGVTQPALFAVEVALFRLLESWGVRPDVLAGHSIGELAAAHVAGVWSLADAVKVVSARAG    114
AT_M3           TGVTQPALFAVEVALFRLLESWGVRPDVLAGHSIGELAAAYVAGVWSLADAVKVVSARAG    112
AT_M7           TGVTQPALFAVEVALFRLLESWGIRPDLLAGHSIGELAAAHVAGVWSLADAVKVVSARAG    112
AT_M10          TGVTQPALFAVEVALFRLLESWGVRPDVLAGHSIGELAAAHVAGVWSLADAVKVVSARAG    112
AT_M11          TGVTQPALFAVEVALFRLLESWGVRPDALAGHSIGELAAAYVAGVWSLADAVKVVSARAG    112
AT_M8           TGVTQPALFAVEVALFRLLESWGVRPDVLAGHSIGELAAAYVAGLWSLADAVKVVSARAG    112
AT_M14          TGVTQPALFAVEVALFSLLESWGIRPDLLAGHSIGELAAAHVAGVWSLADAVKVVSARAG    112
AT_M4           TGVTQPALFAVEVALFRLLESWGVRPGVLAGHSIGELAAAYVAGVWSLADAVKVVSARAG    112
AT_M5           TGVTQPALFAVEVALFRLLESWGVRPDVLAGHSIGELAAAHVAGVWSLVDAVKVVSARAG    112
AT_M12          TGVTQPALFAVEVALFRLLESWGVRPDVLAGHSIGELAAAYVAGVWSLADAVKVVSARAG    112
AT_L            PDVGQPALWAMMVSLSALWRSWGVRPGAVIGSSAGEIPAATVAGALSLEDGARAMALFSQ    117
AT_M1           VDVVQPALWAVMVSLAALWRAYGVEPDAVVGHSQGEIAAAHVAGALSLADAAKVVALRSQ    114
AT_M6           LDVVQPALFAVMVSLAATWRSWGVEPSAVLGHSQGEIAAACVAGALSLEDAARLAVVRSK    114
                    ****:*: *:*     ::*: *  : * * **: ** *:*  ** *.       .
AT_M13          LMGALPEGGTMLSLRADEDTVRDLVAEHRGAVDVAAVNGPESTVVAGDADAVAAIDRAWR    180
AT_M2           LMQALPAGGVMVAVQATEDEIT---SECSEHVGIAAINGPNALVLSGSELEVAALADRLK    173
AT_M9           LMQALPEGGAMVAVQATEAEVL---PELSGTVGIAAVNGPTSVVVSGVAADVEAVAERWR    171
AT_M3           LMQALPAGGAMVAIQGTEAEVA---ADLPETVGIAVVNGPSAVVVSGVAVDVEAVGERWR    169
AT_M7           LMQALPAGGAMVAIQGSEAEVA---SDLPGTVGIAAVNGPSAVVVSGVAADAEAFGERWR    169
AT_M10          LMQALPQGGAMVAIQATEAEVV---ADLPEAVGIAAVNGPSSVVVSGVAADVEAVGERWR    169
AT_M11          LMQALPQGGAMVAIQATEAEV----VDLPETVGIAAVNGPASVVVSGVAADVEAVGERWR    168
AT_M8           LMQALPQGGAMVAIQATEAEV----VDLPETVGIAAVNGPSSVVISGAAADVEAVGERWR    168
AT_M14          LMQALPAGGAMVAIQATEAEVA---AALPGTVGIAAVNGPAAVVISGVAADVEAVGERWR    169
AT_M4           LMQALPQGGAMVAIQATEAEVA---ADLPEAVGIGAVNGPSSVVVSGVAADVEAVGERWR    169
AT_M5           LMQALPQGGAMVAIQATEAEVA---ADLPETVGIAAVNGPSSVVISGAAADVEAVGERWR    169
AT_M12          LMQALPQGGAMVAIQATEAEVA---VDLPETVGIAAVNGPSSVVISGAAADVEAVGERWR    169
AT_L            AQVHLLERGVMLSVLAHADDVRERLTRYDG-LDLAAVHAPGSVLVSGAEDAATELLADLA    176
AT_M1           AILALSGRGGMVSLAVTADEARERVARWDGRMSVAAVNGPRSVVVAGDADALDELMEACA    174
AT_M6           ALTALAGHGGMVSVALGVDAVRELIAPWAGQLAVGVVNGPAAVVVSGDVAALDELVEVCA    174
                    *   * *:::                 : :..::.* : :::*       .
AT_M13          ERGGKTRYLRVSHAFHSPHVDAVLEDFRAVARTVSYGTPTIPIVSTLTGTLLTQEQARDP    240
AT_M2           AQGRKVTGLRVSHAFHSPLMEPMRDDFRRVLQSVSFAAPAIPIVSTLTGAPVDAEELTSP    233
AT_M9           AAGRKVTRLRVSHAFHSPLMEPMLDDFRRVLDDVDFRAPALPIVSTLTGGQASAAELGSV    231
AT_M3           GAGRKVTRLKVSHAFHSPLMDPMLDDFRAVLETVSYEAPTIPIVSTLTGVRATAEELGSP    229
AT_M7           EAGRKVTKLKVSHAFHSPLMDPMLDDFRRVLEEVSYGSPAIPIVSTLTGRVTSAEELASP    229
AT_M10          AAGRKVTGLKVSHAFHSPLMDPMLDDFRAVLEDVSYGPPAIPIVSTLTGRSATAEELAKP    229
AT_M11          AAGRKVTRLKVSHAFHSPLMDSMLDDFRRVLESVSYEAPAIPIVSTLTGVRVTAQELAEP    228
AT_M8           AAGRKVARLKVSHAFHSPLMDPMLDDFRAVLEEVSYAAPAIPIVSTLTGRVASAEELASP    228
AT_M14          AAGRKVTRLKVSHAFHSPLMDPMLDDFRAVLESVSYGSPAVPIVSTLTGARATTEELGSP    229
AT_M4           EAGRKVTKLKVSHAFHSPLMDPMLDDFRRVLEGVSYGAPAIPIVSTLTGVRATTEELGSP    229
AT_M5           AAGRKVTRLKVSHAFHSPLMDPMLADFRAVLETVSYEAPTIPIVSTLTGARATTEELGSP    229
AT_M12          AAGRKVTRLKVSHAFHSPLMDPMLDDFRAVLEEVSYGSPAIPIVSTLTGVRATAEELGSP    229
AT_L            ADGIRARSIAIRIAAHGRQVDSVLDGLRADLAPIRPRPTDLPIYTATTGGRVDPLTLT-A    235
AT_M1           ADEVRARRVPVDYASHSSHVERIEEELAGLLAGLEPVPARVPMLSTVTGEWLTGTEVG-A    233
AT_M6           ENGVWTRRVQADYAPHSHHVERVEAELLEGLADVVPRSGDVPYFSPLTGGWLDTRELD-A    233
                     .  :    * *.  :: :   :      :      :*  :  **
```

```
AT_M13      EHWVRHAREAVRFLDAVRRLREQGVANFLELGPDAVLTAAGRDCVDEG------------    288
AT_M2       DYWVRHVRRPVRFADAVTALAAQGATTMVEFGPGGVLATMGQDSAPDA----VFVPTLR-    288
AT_M9       EYWVRHVRESVRFADAVGTLRAQGADVFLEIGPGGVLTALGQETAAEA----AFVPALR-    286
AT_M3       EYWVRHVRESVRFHGAVTALREQGVDVFLEIGPGGVLSGLGQSNAPEA----TFVPALR-    284
AT_M7       EYWVRHVRESVRFADAVGTLMAEGVATFVEVGPGGTLSALGQESAPDA----GFVPVL--    283
AT_M10      EYWVRHVRESVRFADAVSTLAGDGVGAFVEVGPGGTLSALGRECAPDA----AFVPVL--    283
AT_M11      GYWVRHVRESVRFADAVRALGEDGVGTFVEVGPGGTLSALGRECAPEA----AFVPVL--    282
AT_M8       EYWVRHVRESVRFADAVRTLGEDGVGTFVEVGPGGTLSALGRECAPEA----AFVPVL--    282
AT_M14      EYWVRHVRESVRFADAVGTLTAEGVATFVEVGLGGTLSALGLESAPDA----GFVPVL--    283
AT_M4       EYWVRHVRESVRFADAVGTLTAEGVATFVEVGPGGTLSALGAGSAPD-------------    276
AT_M5       EYWVRHVRESVRYADAVTTLTAEGVATFVEVGPGGTLSALGQESAPDA----RFVPVLR-    284
AT_M12      EYWVRHVRESVRYADAVTTLTAEGVATFVEVGPGGTLSALGPESAPDA----GFVPVLR-    284
AT_L        DHWCRDLRGTADFESATRAALAAGHRLLLEPSPHPVLTRAVQETAEDAGVDAAVVGTLRK    295
AT_M1       DYWYQNLRNTVRFEEGTHGLLDRGVGVFVECSPHPVLTFGVRETLEAADTDSVVVGTLRR    293
AT_M6       AYWYRSLRQPVEFADSVSALVAEGFKTFVEASPHPVLAAGLGELVG---SGGVVAGSLRR    290
             :* :  *  . :  ..      *   ::* .   .*:
```

Table S6. Multiple sequence alignment of ER domain from module2 *dml* biosynthetic gene cluster and active ER domains.

```
ER_EryM4       GSIDGLVLEKVPASQLQPGPGEVRVAVCATGLNFRDVMLALQVYPDAALMGTEAAGVVEA  60
ER_LkmM4       -SLDALAFAAAPDAGRQPGPGEVRVELRSCGVNFRDVLIALGMYPGAAVMGTEGAGVVTA  59
ER_M2          GSLDDLALVPHPDAAAPLAPGQVRVAVRATGLNLHDVRS-------GAEPGSDVAGVVTE  53
ER_GdmM1       GSLENLVLRPDPEATAPLATGQVRIEVRAAGQNFRDVLVALGGVAGQEGLGGEGAGVVTE  60
ER_RapM1       GTLRDLALVPTDTAERPLQSGEVRVDVRAAGLNFRDVVVALGMVDDKRLAGGEAAGVVLE  60
ER_RapM13      GTLQDLALVPTDTAEQPLRPGEVRIDVRAAGLNFRDVVVALGMVNDNRPTGGEAAGVVLE  60
ER_BorM5       GTFDNLTLGVYPHAEKTLADNEVRVAVHAGGLNFHDVVAALGMVEDDLTLGREAAGVVVE  60
                ::  *.:     :      .:**: : : * *::**              * : ****

ER_EryM4       VGEGVTSVEVGDRVLGLFAGAYAERAVTDARLVAPLPPGWSFAEGAAVPIAFTTAYYALH  120
ER_LkmM4       VGPDVTGVAVGDRVMGLFEGAFGPVAVTDHRLVTPVPPGWSTTEAAALPIAFTTAHYALH  119
ER_M2          VGAQVTDLAVGDRVLGLAAKGRGPLAVTDRELLAPMPVGWSFARAAAVPHAFVTAYYALR  113
ER_GdmM1       VGPGVEGLAVGDRVMGLFPRSFGPLAIADARTVAPIPEGWSYATAAGVPVAYLTALYGLR  120
ER_RapM1       VGPEVQDLAPGDRVFGLVGGGFGRSAIADRRMLGVIPDGWSFTTAASVPVVFATAYYGLV  120
ER_RapM13      VGPDVQDLAPGDRVFGVVGGSFGPAAIADRRMLAVIPDGWSFTTAASVPVVFATAYYGLV  120
ER_BorM5       VGDAVPDLTPGDHVMGILSSGFGPLAVTDHRYLARMPEGWTFAQAASVPAAFLTAYYGLC  120
               **   *  .:   **:*:*:    .  .   *::*  . :   :* **: :  .*.:*  .: ** *.*

                             HAAAGGVGMA
ER_EryM4       DLAGVQRGQTVLVHAAAGGVGMAAVSLALHAGADVLATASPAKHDAVQARGLPADQVRSS  180
ER_LkmM4       DLAGVRPGQAVLVHAAATGVGMAAVRLARLAGAEVFATASPAKQGVLRSLGLDDDHIASS  179
ER_M2          DLARLSRGQSVLVHAPTGDVGAAAVQLARHWGAEAHTTGTGG------------------  155
ER_GdmM1       DLGTVQPGETVLVHAAAGGVGMAAVQLARHFGATVYATAHPSKHHVLTALGVPEGHLASS  180
ER_RapM1       DLAGLSAGESVLIHAAAGGVGMAATQIARHLGARIYATASTGKQHILREAGLEDTHIADS  180
ER_RapM13      DLAGLSEGESVLIHAAAGGVGMAATQIARHLGARIYATASTGKQHILREAGLEDTHIADS  180
ER_BorM5       DLGGIRAGDRVLIHAAAGGVGMAAVQIARHLGAEVFGTASPRKWGALRALGLDDAHLSSS  180
               **. :  *: **:** : .** **. :*   **    *.

ER_EryM4       RTSGFAEAFLGHTRGRGVDVVLNSLTGPLLDETIRLVAPDGVVVELGKADLRDPADVAA-  239
ER_LkmM4       REPGFGRRFRAVRGGRGMDVVVNSLTGALLDESAELLAEGGAFVEMGKTDLRDADRFRG-  238
ER_M2          ---------------RDADVVLDA------------------------------------  164
ER_GdmM1       RDLGFASAFP------ALDVVLNSLTGEYVDASLGLLGTGGRFVEMGKNDIRDPASVAAA  234
ER_RapM1       RTLSFQETFLNNTHGQGVDVVLNSLSGDFVDASLDLLPRGGRFIEMGKTDIRDPHQVTAD  240
ER_RapM13      RTLSFQETFLNNTHGQGVDVVLNSLSGDFVDASLDLLPRGGRFVEMGKTDIRDPHQVTAD  240
ER_BorM5       RTLDFEQEFLDATDGRGVDLVLNSLAREFVDASLRLMPGGGRFVDMGKTDIRRPEQVAED  240
                                 *:*:::

ER_EryM4       ----RYLPFDLG-EVGPDRLGEILRTVVDLLAGGRVPPVPVSARPLADLRAALTEMSRGG  294
ER_LkmM4       ----RYLPFDLA-EAGADRLGAILAEVASLAAGGELGRQTVTAWPLQRASAALQHMSTGR  293
ER_M2          --------AYAGKRPDPDRFQEILTEVLALFDAGALNPLPVRAWDVRRAPEAFRHLEQAA  216
ER_GdmM1       HPGVGYQAFDLGGDAGPDRIRELLAELVELFEAGRIEPLPIRHWDVTQAPTAFRWMSQGR  294
ER_RapM1       RPGTTYQAFDLM-DAGPDRLREIITELLTLFTQGVLLPLPVQAWDIRQARDAFSWMSRAR  299
ER_RapM13      RPGTTYQAFDLM-DAGPDRLREIITELLTLFTQGVLLPLPVQAWDIRQARDAFSWMSRAR  299
ER_BorM5       HGGVAYQAFDLV-EAGPQRTGEMLAEIVRLFQAGAFRPLPITQWDVRRAPEAFRHISQAK  299
                              . :*   ::  :  *   * .    :    :     *:  :. .
```

Table S7. Multiple sequence alignment of DH domains from *dml* biosynthetic gene cluster. Conserved HXXXGXXXXP, GYXYGPXF, DXXX(H/Q), and LPFXW motifs are highlighted.

```
                                                    HXXXGXXXXP
DH_M2       HALLRAAVALPDSDGVVFTGRLSTRTQPWLADHGVGGRVLFPGTGFLDLALHAARHCGLD     60
DH_M6       HPVLVAVTELADSEGLVFSGRLSLRTHPWLTDYRVLGAALLPGAAYADLVLRAGDHVGCA     60
DH_M14      HPLLGAAVELADGQGLVCTGRLGTDTHPWLADHAVGQTVLLPGTAFADIALAAGGRLGLD     60
DH_M13      HPLLGAAVTLADEEGALLTGRLSLATHPWLADHTVGGVVVVPGAALVELAVRAGDQVGLD     60
DH_M12      HPLLGAVIDTADSDGGLFTGRLSLESHPWLAEHAIQGSVLLPGTAFLDLAIHAGDQVGCD     60
DH_M11      HPLLGASVALAGTDGALLTGRLSVQSHPWLADHAVMGSVLLPGTAFLDLAIRAGDQVGCD     60
DH_M8       HPLLGASVGLAGTDGVLLTGRLSVQTHPWLAEYVVQGSVVLPGSAFVELAVRAGDQVGCD     60
DH_M4       HPLLGASVGLAGTDGVLLTGRLSVQSHPWLADHEVLGSVVLPGSAFVELAVRAGDQVGCD     60
DH_M7       HPLLGASVALAGTDGVLLTGRLSVQSHPWLADHEVLGSVVLPGSAFVELAVRAGDQVGCD     60
            * :* *     . :* : :***.  ::***::: :   .:.**:.  ::.: *. : *

DH_M2       TVEELTLHAPLVLPERGGLALRVAVGAETDGG-RSIRVHARAEDA---------------    104
DH_M6       SLDDLVLETPLVLPERDGVQVRLSLDGPDASGRRAFTVDSRG------------------    102
DH_M14      EVEELTLAAPLVLPERGGVRLRVTVGGDDGDGRRTLIVDSRPDTPNTPGTPDTSAKEASE    120
DH_M13      LVEELTLAAPLVLPEDGEVRLQVSVGAPDASGRRTVSAYARPDDA---------------    105
DH_M12      LVEELTLEAPLVVPERGGVLVQVQVGVADESGRRTVTVHAR---G---------------    102
DH_M11      LVEELTLEAPLVLPESGAVRVQVWVGAEDASGRRELTFHSSTGDM---------------    105
DH_M8       LVEELTLEAPLVLPESGAVRVQVWVGAEDSSGRRELSFYSSAGEV---------------    105
DH_M4       LVEELTLEVPLVLPEGGAVRVQVWVGAEDPSGRRELSFYSSAGDV---------------    105
DH_M7       LVEELTLEVPLVLPEGGAVRVQVWVGAEDPSGRRELSFYSSAGDV---------------    105
              :::*.* .***:** . : ::: :.    .* *  .    :

                                                                 GYXYGPX
DH_M2       ERDEPWTEHASGTLTTGDAPATDAPATDLTAWPPEGATPMDVDDVYDRLT-ELGYGYGPV    163
DH_M6       DGGGEWTRHATGALAAGAE----PVTDDLVAWPPAEAEPVDLDDHYVTVAASSGLDHGPA    158
DH_M14      VDEADWIRHATGFLTTATP----TAPAPLTQWPPAGAEPVGIDGFYEGLE-EAGFAYGPA    175
DH_M13      PDDQPWTRHAFGTLTTGDD----APAHRAEAWPPEGAERIDIEGRYDDLA-ASGLGYGPA    160
DH_M12      DDGDVWVRHASGVLVGGAD----EPSADAGAWPPAGAEAVGLDGLYDRMA-DGGFGYGPL    157
DH_M11      EDSRSWTRHATGVLRAGGR----SEGAPLVAWPPAGAEVVDLDGFYEGMA-EGGFGYGPV    160
DH_M8       DEGRVWTRHATGVLSEGQR----VGGTSLVAWPPAGAEVVDLDGFYEGMA-EGGFSYGPV    160
DH_M4       DEGRVWTLHATGVLGEGGR----SDGVSLVAWPPPGAEVVDLEGFYDR----ADFAYGPV    157
DH_M7       DEGRVWTLHATGVLGEGGR----SDGVSLVAWPPPGAEVVDLEGFYDR----ADFVYGPV    157
                 *   ** * *   .                ***   *   :.::. *        .   :**

            F                                  DXXXH/Q               LP
DH_M2       FRALRAAWRLGRDVYVEVALEDAS--GAEGFSVHPALLDAALHAPVLRALDETGSGPRLP    221
DH_M6       FQGLRAAWRKGDEVFAEVALDDGL--ADDGYGLHPALLDGALHAIGLGVSA-----PGLP    211
DH_M14      FQGLRTAWRGDGAVYAEIELDEAQDRDAAAFGLHPALLDAALHACMLGGLVEDAGRPRLP    235
DH_M13      FRGLRTAWRRGDEVFVEVELDDQD--AAGAFGLHPALLDSALHAIGLGGFVADAERLHLP    218
DH_M12      FQGLRSAWRKGDEVFAEVALPDGV--EAGGFGLHPALLDAALHAIGLMGGADGP--GRLP    213
DH_M11      FQGLRAAWRAGDEVFAEVALPEGV--KAEGFGLHPALLDAALHATGLTGAADAP--GKLP    216
DH_M8       FQGLRAAWRTGDEVFAEVVLPEEV--KAEGFGLHPALLDAAMHAMGLMGEAEGP--GRLP    216
DH_M4       FRGLRAAWRTGDEVFAEVVLPEGV--QAEGFGLHPALLDAALHAMGLMGEVEGL--GRLP    213
DH_M7       FRGLRAAWRTGDEVFAEVVLPEGV--QAEGFGLHPALLDAALHAVAVGDADDDT--GRLP    213
            *:.**:***  .   *:.*: * :        .:.:******.*:**   :              **

            FXW
DH_M2       FGFTGVRLHAVGTSTLRVRWSPTGQDEMSLAVADPAGRPVATVESLVMRPAE----        273
DH_M6       FSWAGVRLLATGATTLRVRLTPVGVDAVAVLVTDGTGQPVAAVDELSLRPLSADQL        267
DH_M14      FSWSGVRWHATGATTARVRLTPAGPDAVSLELADAQGNPLATVASLVLRPIAA---        288
DH_M13      YSWRGVRLHSGGASALRGRLSPAGASGVSISLTDGTGAPVASVEALSLRPLAADG-        273
DH_M12      FSWSGVRLHASGATVLRVRLAPTASDGVSLTVTDAAGAPVATIDSLVLRPVS----        265
DH_M11      FSWSGVRLHASSATALRVRLSPTGPDGVSLTVADGSGAPVATIDSLVLRPAAPA--        270
DH_M8       FSWSGVRLHASGATVLRVRLAPTSADGVSLAVADGTGAPVATVDSLVLRPAA----        268
DH_M4       FSWSGVRLHASGATVLRVRLAPTSADGVSLAVADGTGAPVATVDSLVLRPVSL---        266
DH_M7       VSWSGVRLHASGATVLRVRLSPTGSDAVSLTVADGAGAPVASVDSVVLGPVSSQ--        267
             .: ***  : .::. * * :*.. . ::: ::*  * *:*::  : : *
```

Table S8. Multiple sequence alignment of KR domains from *dml* biosynthetic gene cluster.

```
KR_M9           GTVLVTGGTGALGTHIAKWLI-SKGAEHLLLTSRRGPKAAGAEELRAELTALGARVTIAA    59
KR_M2           -TVLVTGGLGTLGALVARHLVTDRGVKHLVLAGRKGLATDGAPELRDELAALGADVSVAA    59
KR_M6           -TVLITGGLGVLGGVTARHLVTRHGVRNLVLTGRRGPATPGAQQLRAELEELGARVTVVA    59
KR_M12          GAVLVTGASGMLGGLVARHLVVRHGVRRLVLASRRGQV----GALYDELVGLGAEVAVVA    56
KR_M13          GAVLVTGASGMLGGLVARHLVVRHGVRRLVLASRRGQV----GDLCDELVGLGAEVAVVA    56
KR_M14          GTVLVTGATGMIGGLVTRHLVTEHGVRHLVLASRRGDAAPGADALRAELTGLGADVTLAA    60
KR_M8           GTVLLTGPTGPLGSLVARHLVTERGVRNLLLVSRHEADAEGAAQLRDELAAQGAEVVLAA    60
KR_M7           GAVLITGATGTLGGLVARHLVAERGVRSLLLVSRRGAEAEGAAELRDELTAQGAEVVFAA    60
KR_M4           GAVLITGATGTLGGLVARHLVAERSVRSLLLVSRRGGEAEGAAELRDELTAQGAEVVFAA    60
KR_M11          GAVLITGATGTLGGLVARHLVAERSVRNLLLVSRRGADAEGAAELRDELAAQGAEVVFAA    60
KR_M1           GTVLITGGTGAIGGHVARWLA-GNGAEHLVLTSRRGADAPGARELEAELTALGVRTTVAA    59
KR_M5           GTVLVTGGLGALGATAARWLA-ANGAEHVVLASRRGRAAEGAADLEAELVGLGARVTLAA    59
KR_M3           GTVLVTGGTGALGGRVARWLA-ERGAEHLVLASRRGAEAEGVADLEAELVGLGARVTVAA    59
KR_M10          GTVLVTGGLGALGGHVARWLA-ERGAEHLVLASRRGAEAEGATELEAELVELGARVTFAA    59
                 :**:**  * :*   :: *   .... ::*..*:         *  **   *. . ..*

KR_M9           CDVADRDALADLLASVPA-EFPLTAVVHAAGVLDDGVLSALTPDRL---DAVLRPKVDAA    115
KR_M2           CDVADRDAVAALLAA--L-DRPLGAVVHTAGVVDDGVLESLTPERI---HRVFAPKVEGL    113
KR_M6           CDVSERAEVAALL----A-EHPVTAVVHTAGVLDDGVIESLTPEHV---DRVLGPKADAA    111
KR_M12          CDVAQRDAVAALL----A-EHPVTAVVHTAGVLDDGTIGSLTPERI---DTVFGPKVDGA    108
KR_M13          CDVAQRDAVAALL----A-EHPVTAVVHVAGVLDDGVIDSQTPERI---DTVFGPKADGA    108
KR_M14          CDVTDRAALQELLAAVPE-DHPLTGVVHSAGVLDDGVIGSLTPERI---DTVFRPKVDAA    116
KR_M8           CDTADREALAALL----A-AHQVSAVVHTAGELDDGTIGSLTPERI---DTVFRPKVDAA    112
KR_M7           CDVADREAVAALL----A-EHPVTAVVHTAGVLDDGVIGSLTPERI---GTVFRPKVDAA    112
KR_M4           CDVAEREAVAALL----A-AHPVTAVVHTAGVLDDGVIGSLTPERI---GTVFRPKVDAA    112
KR_M11          CDVADREALAALL----A-EHPVTAVVHTAGVLDDGVIGSLTPERI---GTVFRPKVDAA    112
KR_M1           CDAADRGALADLFARLDADGTPVRSVFHAAGTVPSLPLADTDTEDL---AYALAAKAAGA    116
KR_M5           VDVADKDGVAALLGGLAAAGDPVRAVVHAAGLNGSVPI---AETDLAWFADVVTAKVAGA    116
KR_M3           CDATDRDAVAALLAGLPS----LTAVVHTAGVERPAALADLDPDDLTDFAHVLAAKAGSA    115
KR_M10          CDMTDRESVAALLAGVPA----LDAVVHAAGIERSALLTDLDPSDLGGFADVLAAKAVGA    115
                 * :::  :  *:         : .*.* **      :       :     ..  *. .

KR_M9           VNLDELTRGHELSAFVMFSSTSGALGGPGQANYAAANTFLEALADRRAAAGLPATAVAWG    175
KR_M2           LHLDELTRDADLSAFVVFSSAAGILGSAGQASYAAANTALDALVRRRHALGLPGVSLAWG    173
KR_M6           RHLHELTLGTDLTAFVLFSAVAGVFGSPGQGNYAAANAYLDALAQHRRAAGLPATSLAWG    171
KR_M12          WHLHELTREMGLSAFVLFSSVTGTLGSPGQGNYAAANAFLDALARHRRAQGLAATSLAWG    168
KR_M13          RHLHELTREMDLSAFVLFSSAAGTFGNPGQSNYAAANAYLDALAQHRRAAGLPATSLAWG    168
KR_M14          WHLHELTADLGLSAFVLFSSASGVFGTPGQGNYAAANTFLDALAQHRRAAGLPATSLAWG    176
KR_M8           WHLHELTADLDLSAFVLFSSATGLFGAPGQGTYAAANTFLDALAQHRRAAGLPATSLAWG    172
KR_M7           WHLHELTADLDLSAFVLFSSAAGVLGGPGQGNYAAANAFLDALAQHRQAAGLPATSLAWG    172
KR_M4           WHLHELTAGLGLSAFVLFSSASGVFGAPGQGNYAAANTFLDALAQHRRAQGLPAASLAWG    172
KR_M11          WHLHELTADLGLSAFVLFSSASGVFGAPGQGNYAAANTFLDALAQHRRAQGLPATSLAWG    172
KR_M1           VHLDELCAGRDLDAFVLFSSGSAVWGSGELGAYGAANAFLDGLAQRRAAEGLPATSVSWG    176
KR_M5           AHLHELLSDTPLDAFVSYSSIAGTWGSGGQAAYSAANACLDALAEHRTARGLPGTAVAWG    176
KR_M3           RILHELLADRPLDAFVLFSSIAGVWGSGGQAAYGAANAYLDALATHRRAAGLPATAVAWG    175
KR_M10          RHLHELLGDTPLDAFVLFSSISGVWGSGAQSAYGAANAYLDALAEHRRAAGLPATAVAWG    175
                  *.**     * *** :*: :.  *    . *.***: *:.*. :* * ** ..:::**

KR_M9           PWG- 178
KR_M2           MWA- 176
KR_M6           LWAE 175
KR_M12          LWA- 171
KR_M13          LWA- 171
KR_M14          LWE- 179
KR_M8           PWT- 175
KR_M7           LWAE 176
KR_M4           LWE- 175
KR_M11          LWE- 175
KR_M1           MWA- 179
KR_M5           PWA- 179
KR_M3           PWG- 178
KR_M10          PWA- 178
                 *
```

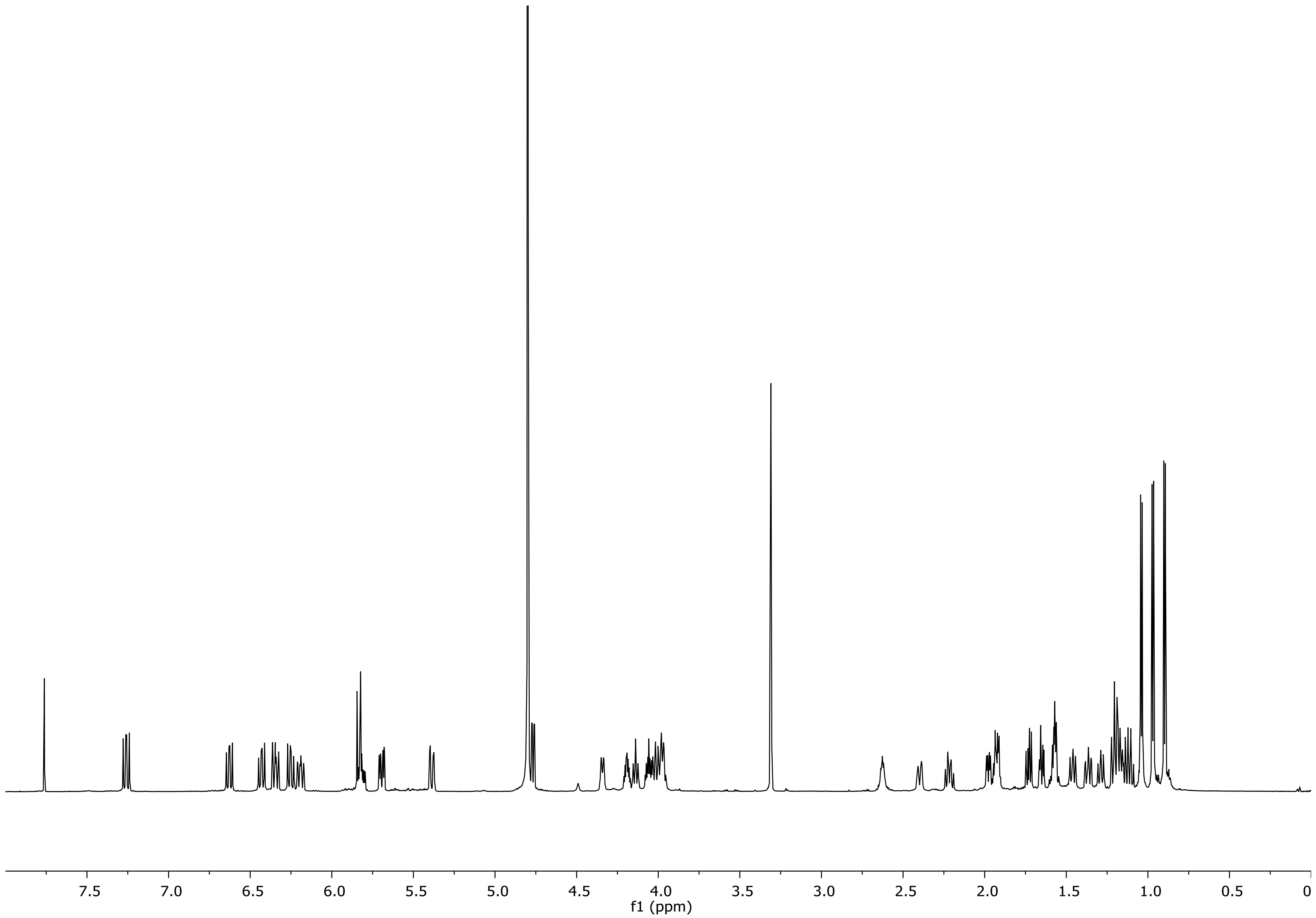

Figure S3. ^{1}H NMR spectrum of demurilactone A in $CD_3OD/CDCl_3$ (3:1).

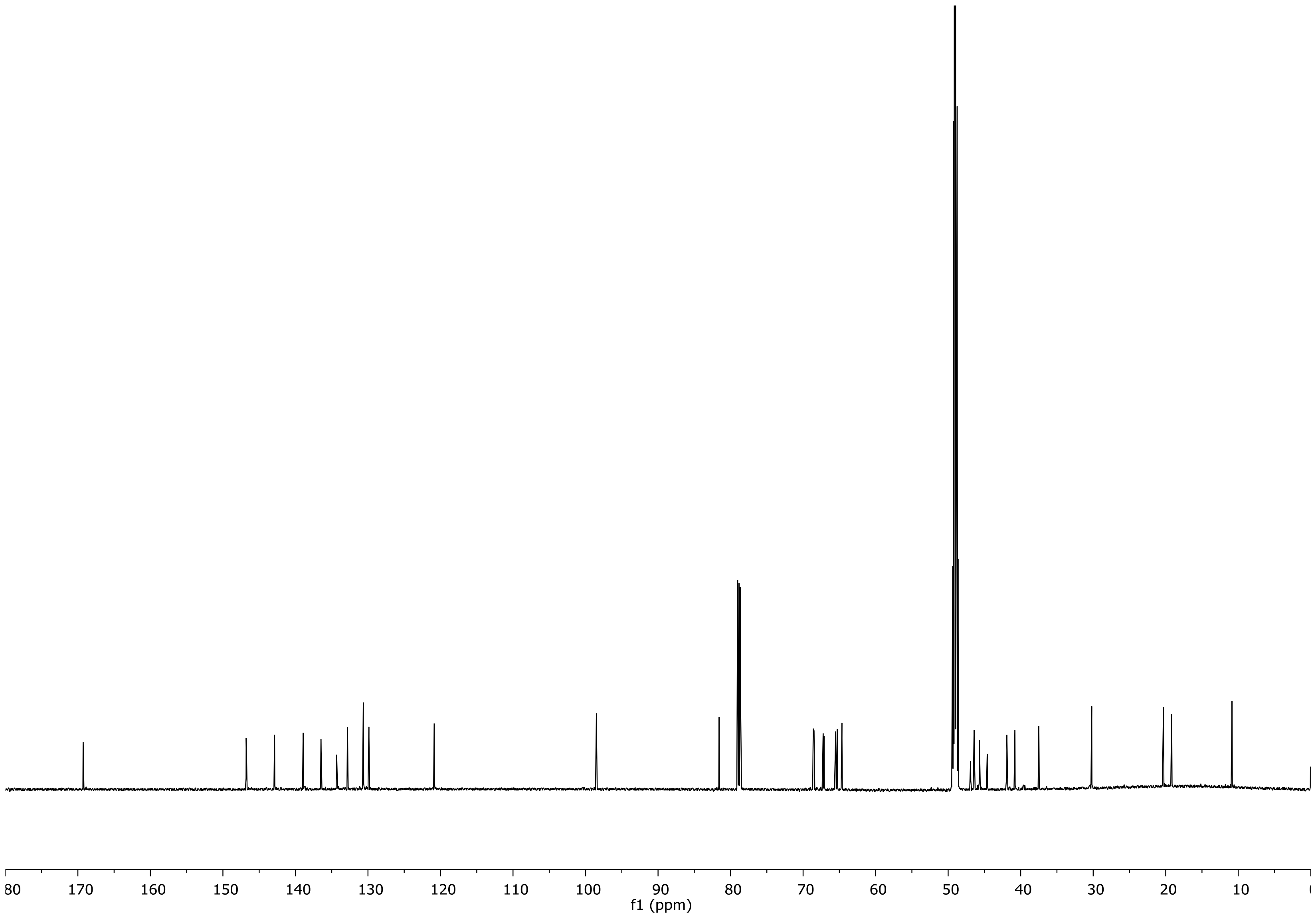

Figure S4. ^{13}C NMR spectrum of demurilactone A in $CD_3OD/CDCl_3$ (3:1).

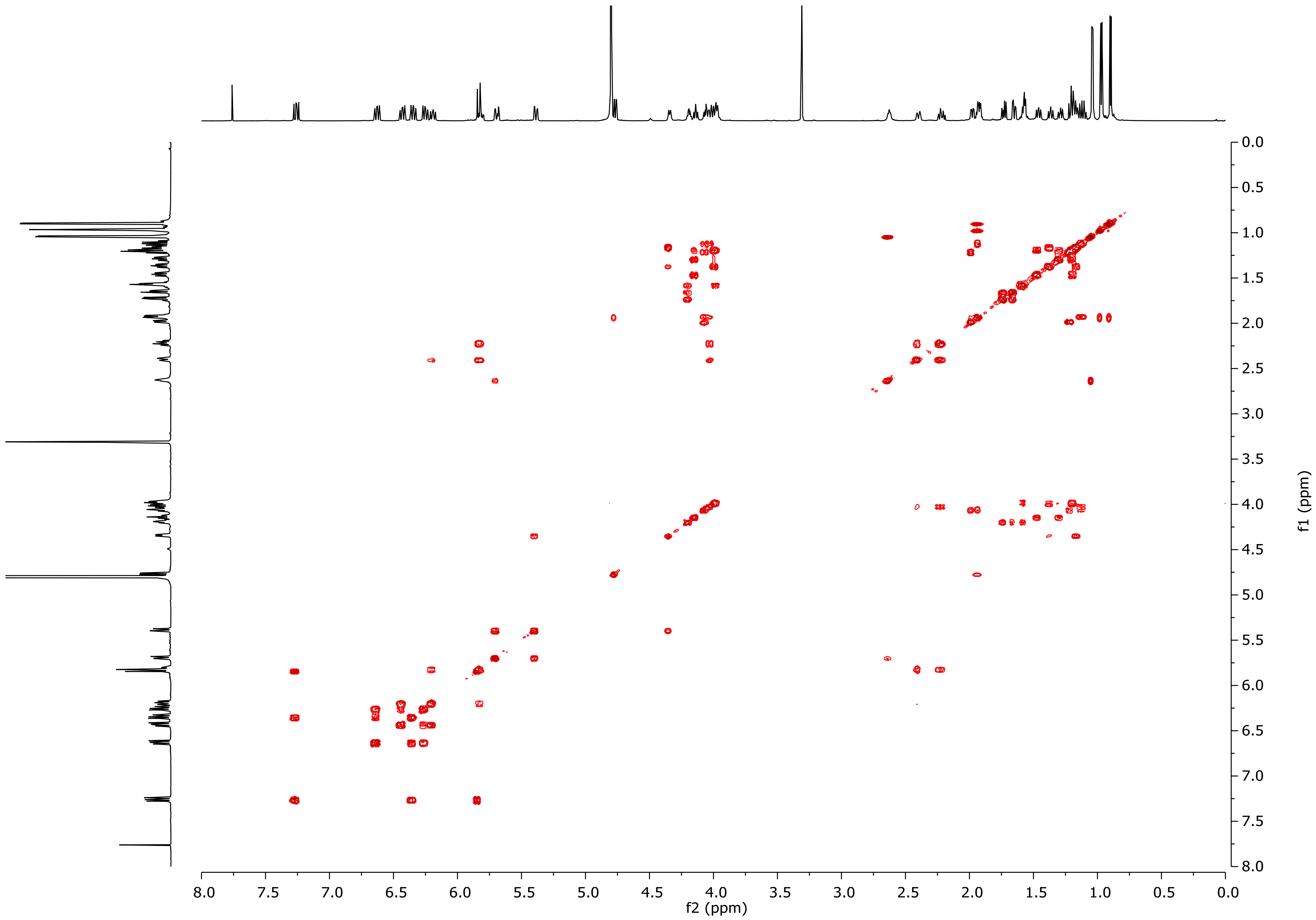

Figure S5. COSY spectrum of demurilactone A in $CD_3OD/CDCl_3$ (3:1).

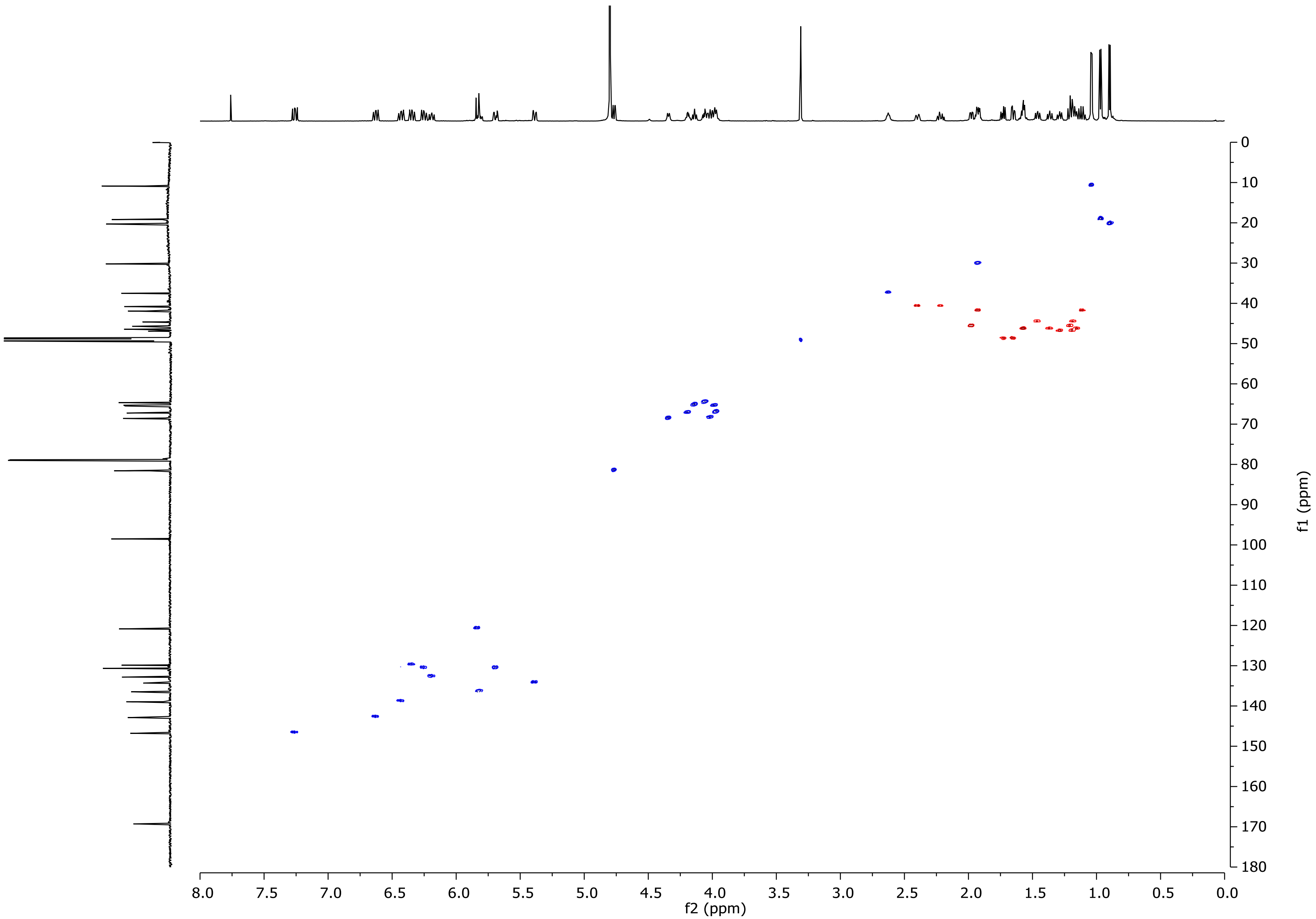

Figure S6. HSQC spectrum of demurilactone A in $CD_3OD/CDCl_3$ (3:1).

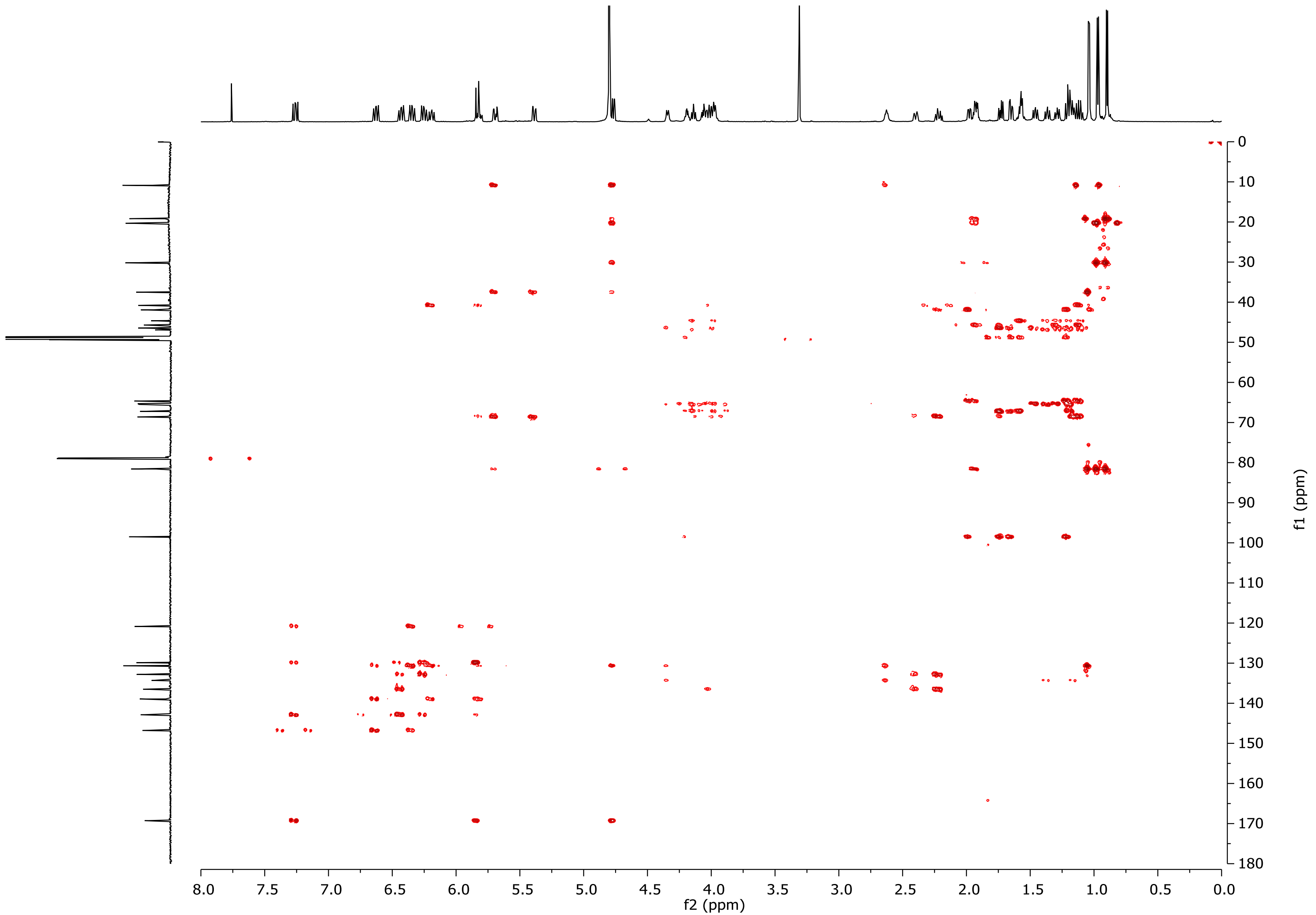

Figure S7. HMBC spectrum of demurilactone A in $CD_3OD/CDCl_3$ (3:1).

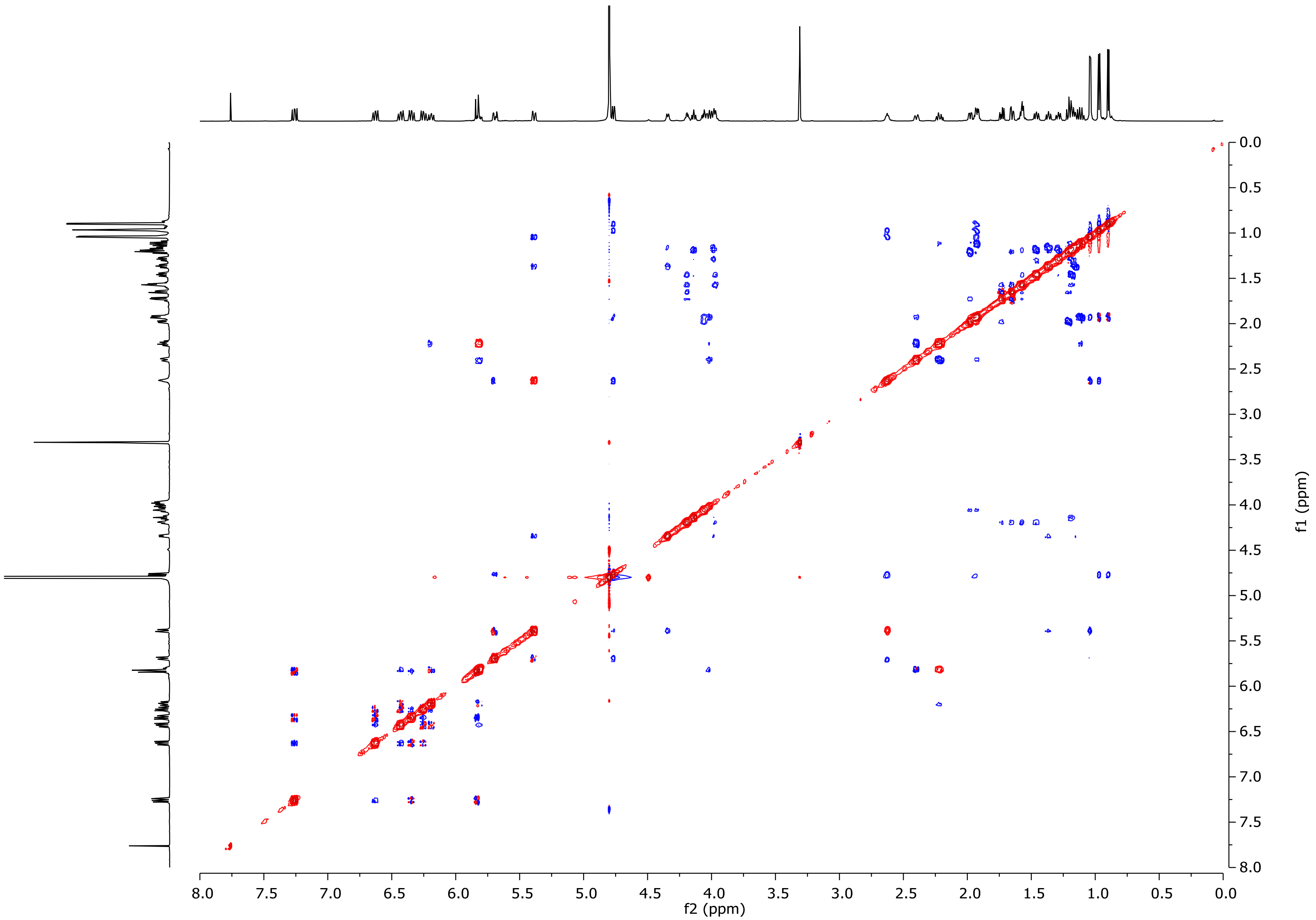

Figure S8. ROESY spectrum of demurilactone A in $CD_3OD/CDCl_3$ (3:1).

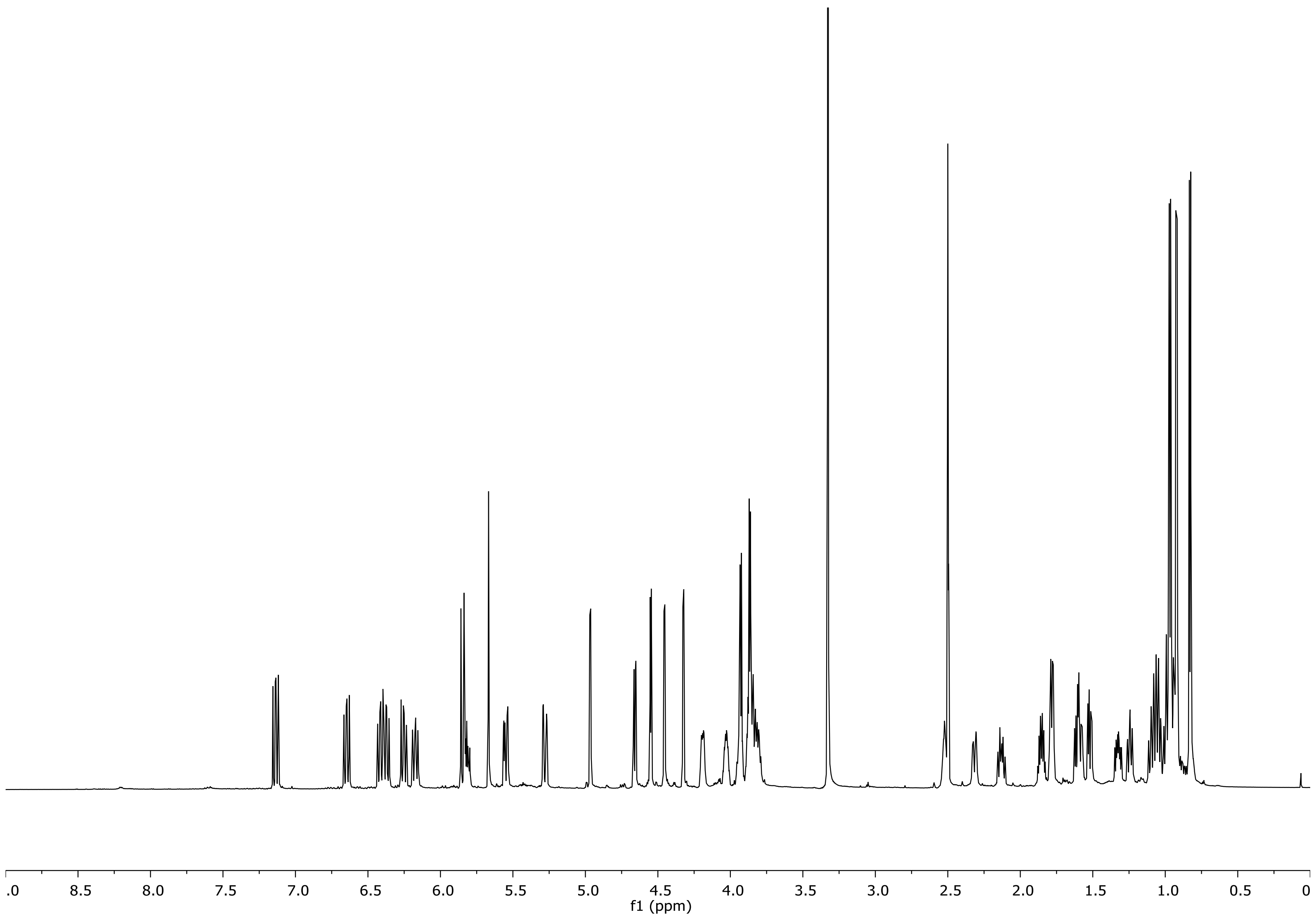

Figure S9. ^{1}H NMR spectrum of demurilactone A in DMSO-d_6.

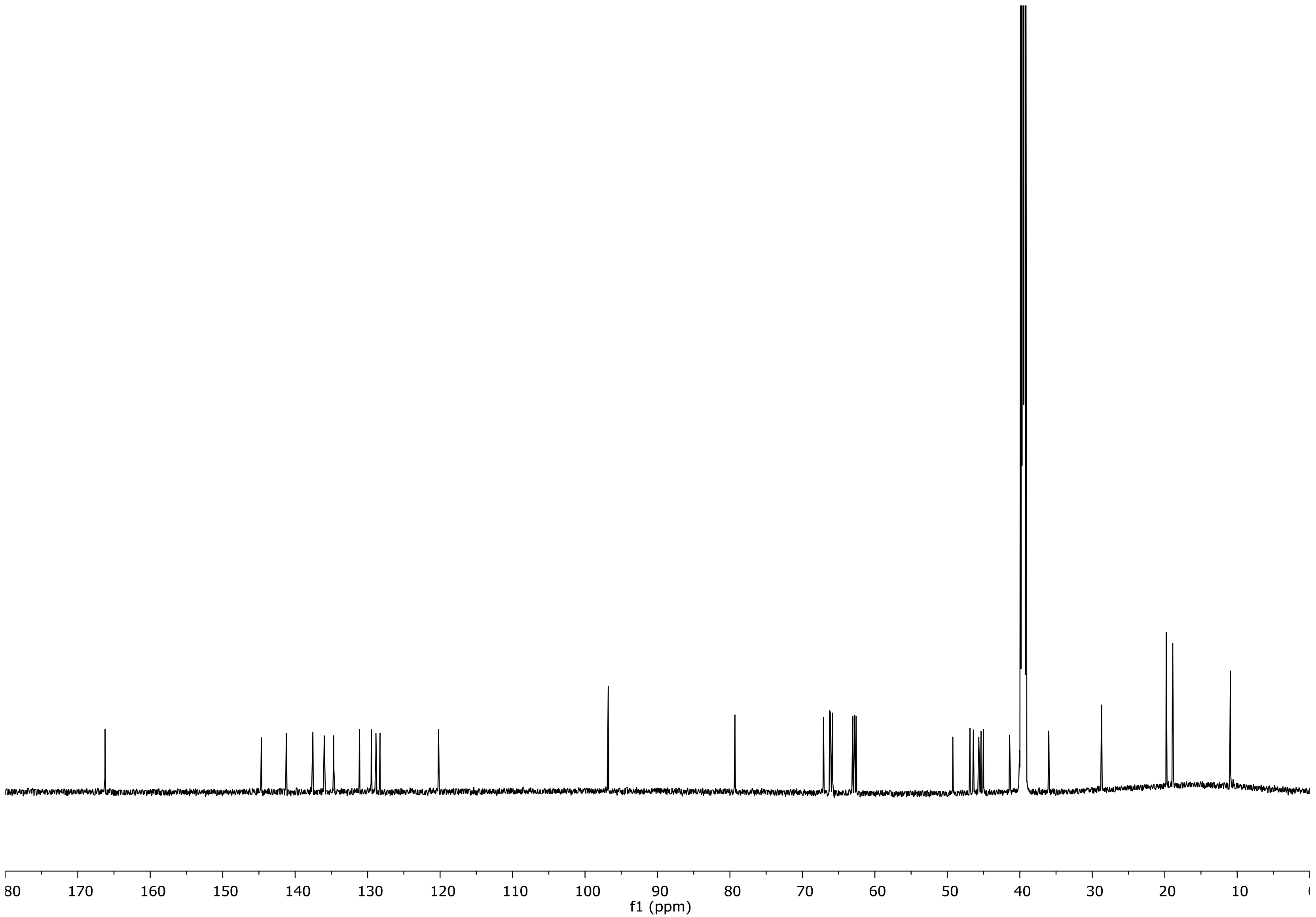

Figure S10. ^{13}C NMR spectrum of demurilactone A in DMSO-d_6.

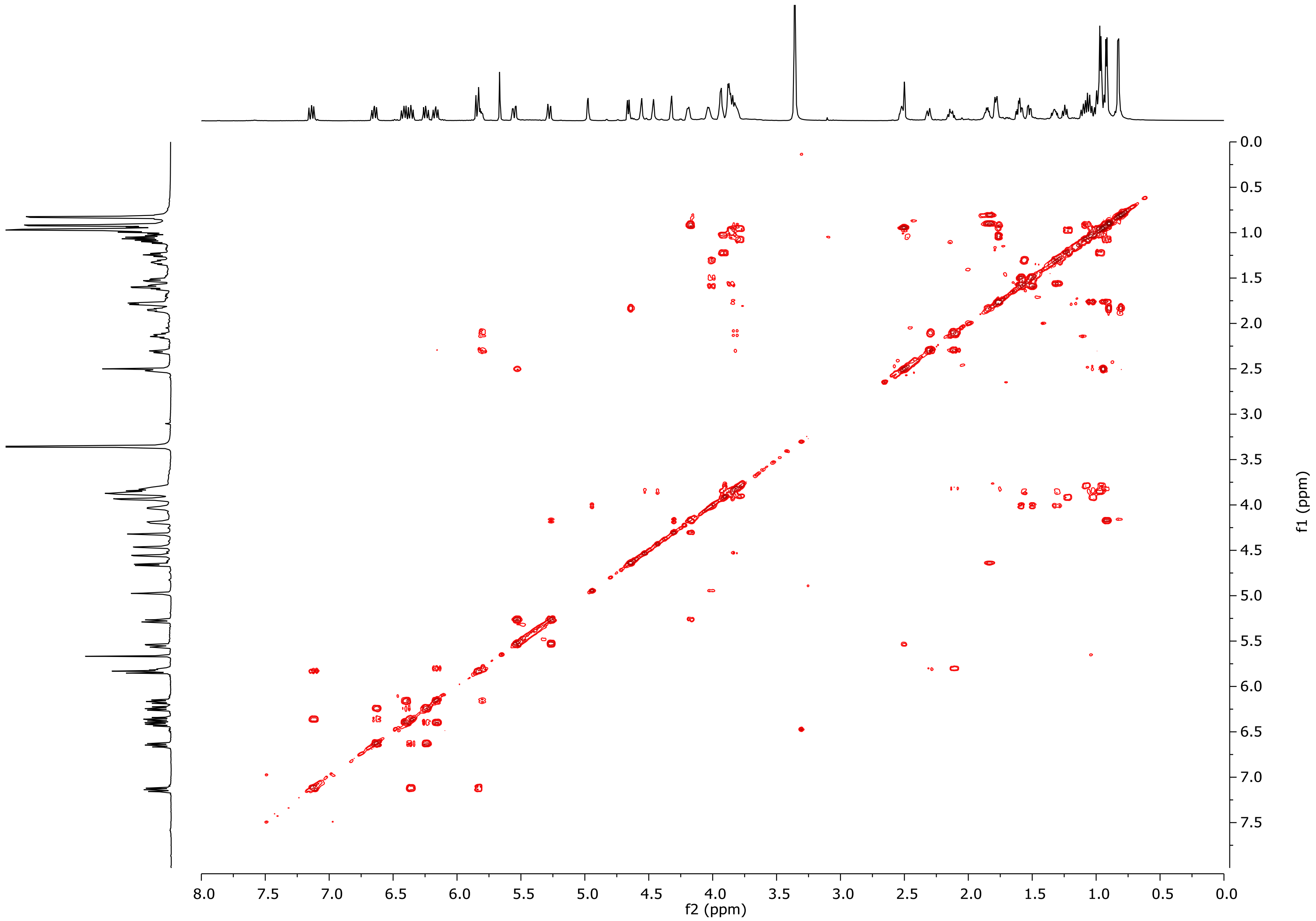

Figure S11. COSY spectrum of demurilactone A in DMSO-d_6.

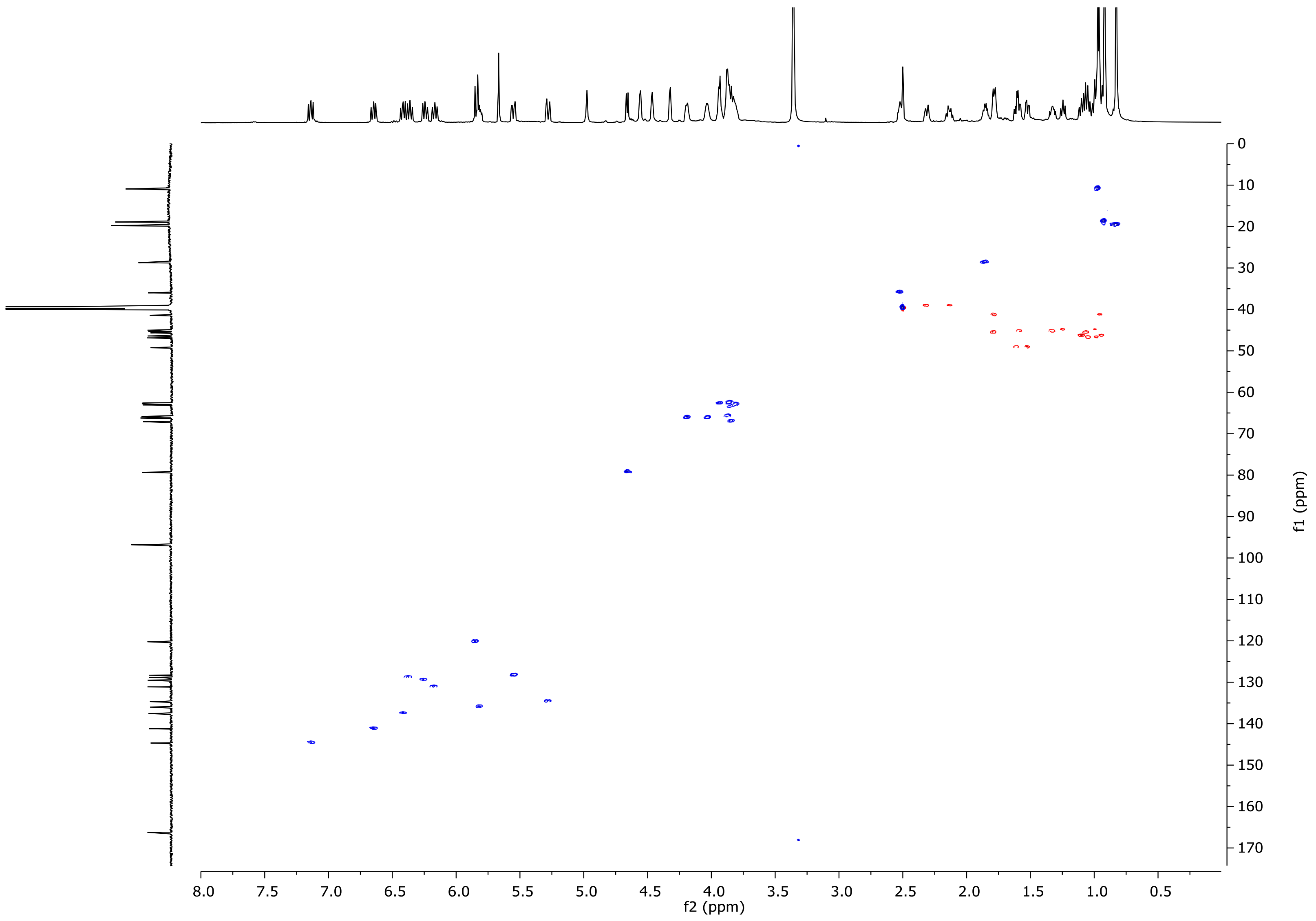

Figure S12. HSQC spectrum of demurilactone A in DMSO-d_6.

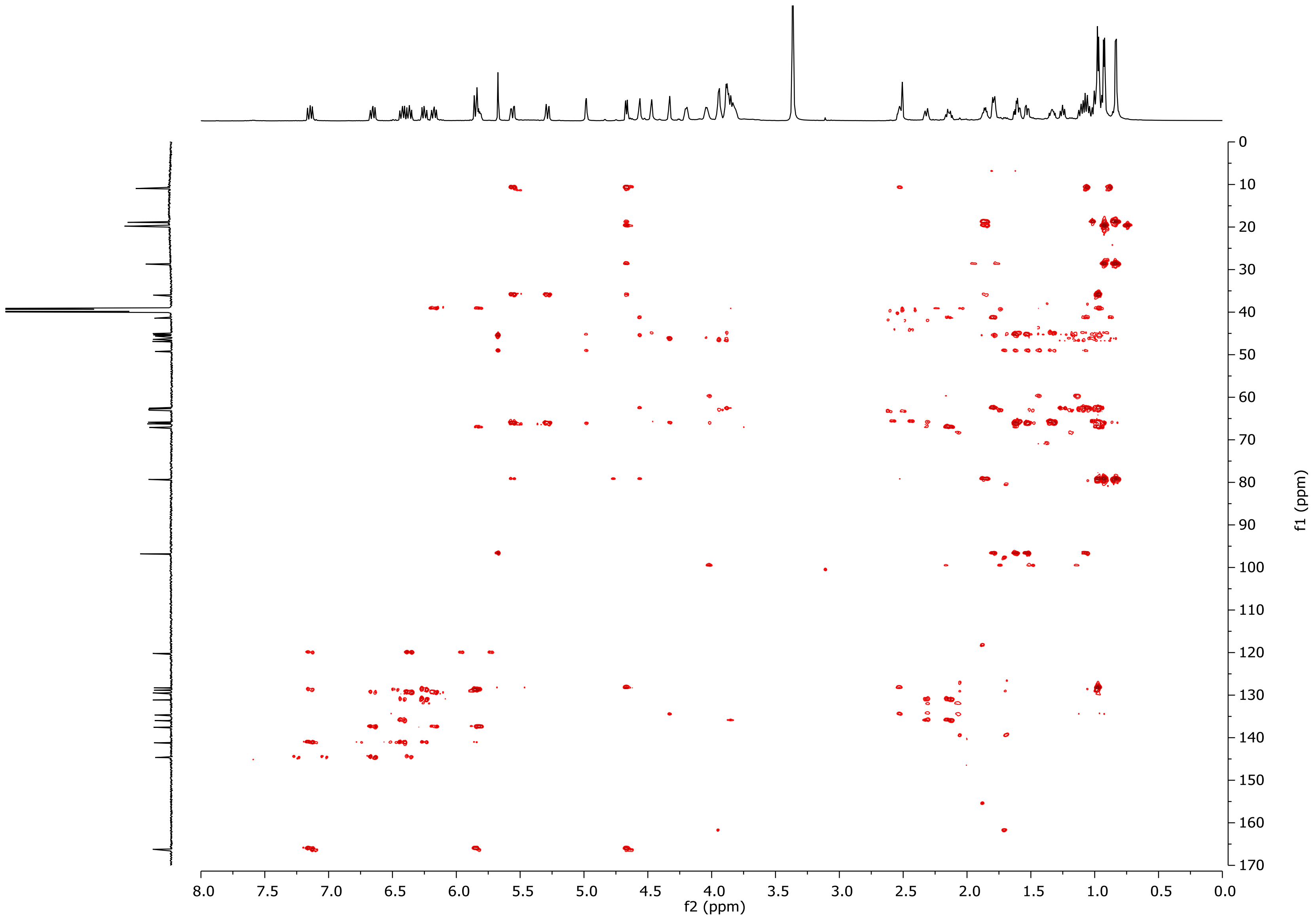

Figure S13. HMBC spectrum of demurilactone A in DMSO-d_6.

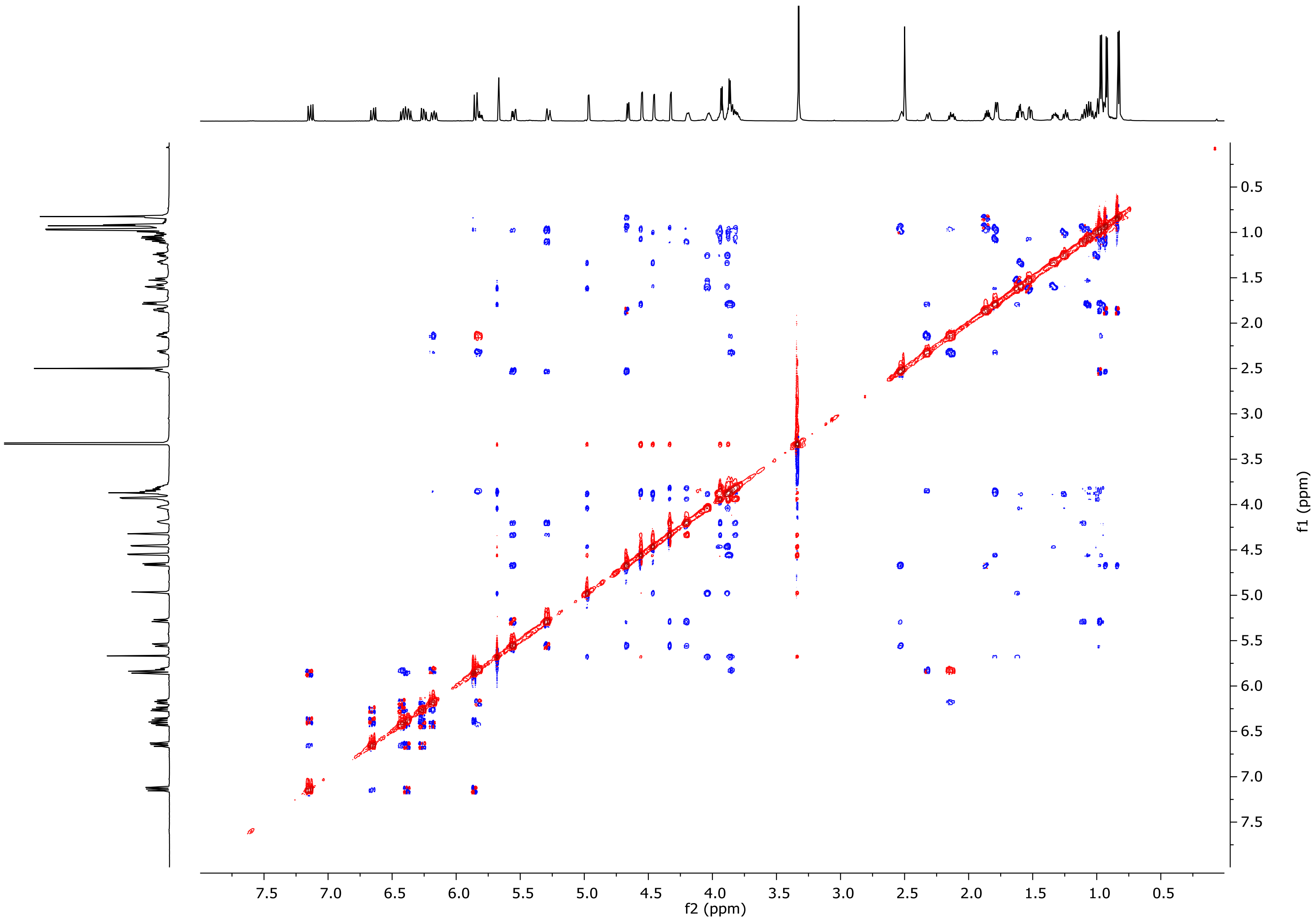

Figure S14. ROESY spectrum of demurilactone A in DMSO-d_6.

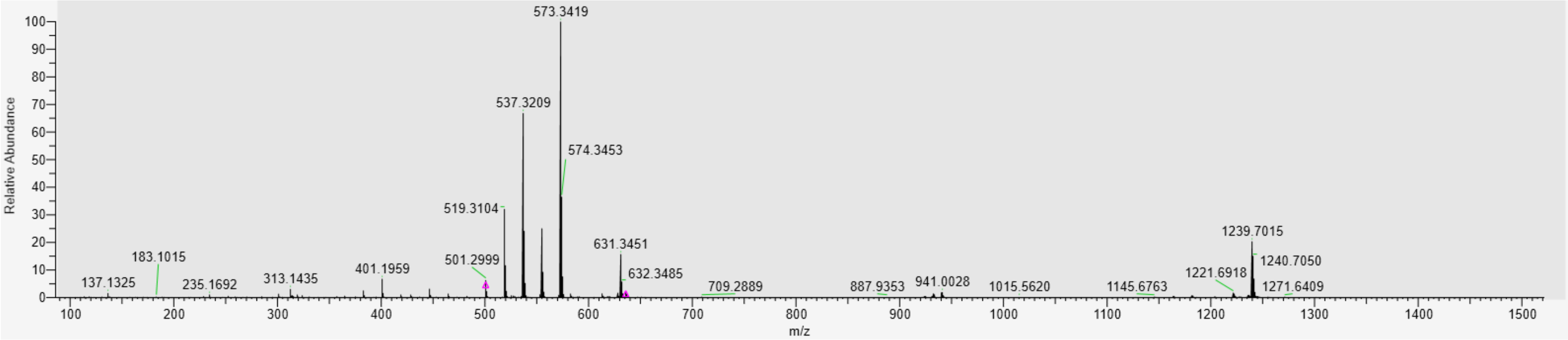

Figure S15. HR-MS spectrum of demurilactone A showing $[M+Na]^+$ = 631.3451, $[2M+Na]^+$ = 1239.7015, and masses corresponding to consequence loss of water from demurilactone A.

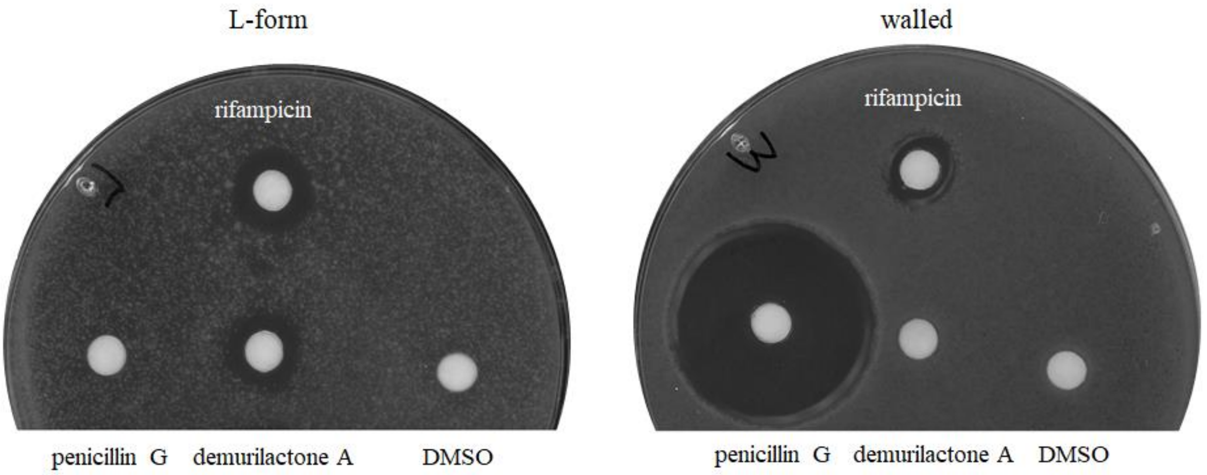

Figure S16. Comparative growth inhibitory experiments of penicillin G (50 µg), rifampicin (0.5 µg), demurilactone A (64 µg), and DMSO (5 µl) against L-form (Left panel) and walled (Right panel) *Bacillus subtilis*.

Table S9. Zone of inhibition (diameter; cm) of three replicates of penicillin G (50 µg), rifampicin (0.5 µg), demurilactone A (64 µg), and DMSO (5 µl) against walled and L-form *Bacillus subtilis*. Diameter of the discs is 0.6 cm.

	Walled *Bacillus subtilis*			L-form *Bacillus subtilis*		
Compound	Plate 1	Plate 2	Plate 3	Plate 1	Plate 2	Plate 3
Penicillin G	3	3	3	0	0	0
Rifampicin	1	1	0.9	1.3	1.2	1.3
Demurilactone A	0	0	0	1.2	1.2	1.2
DMSO	0	0	0	0	0	0

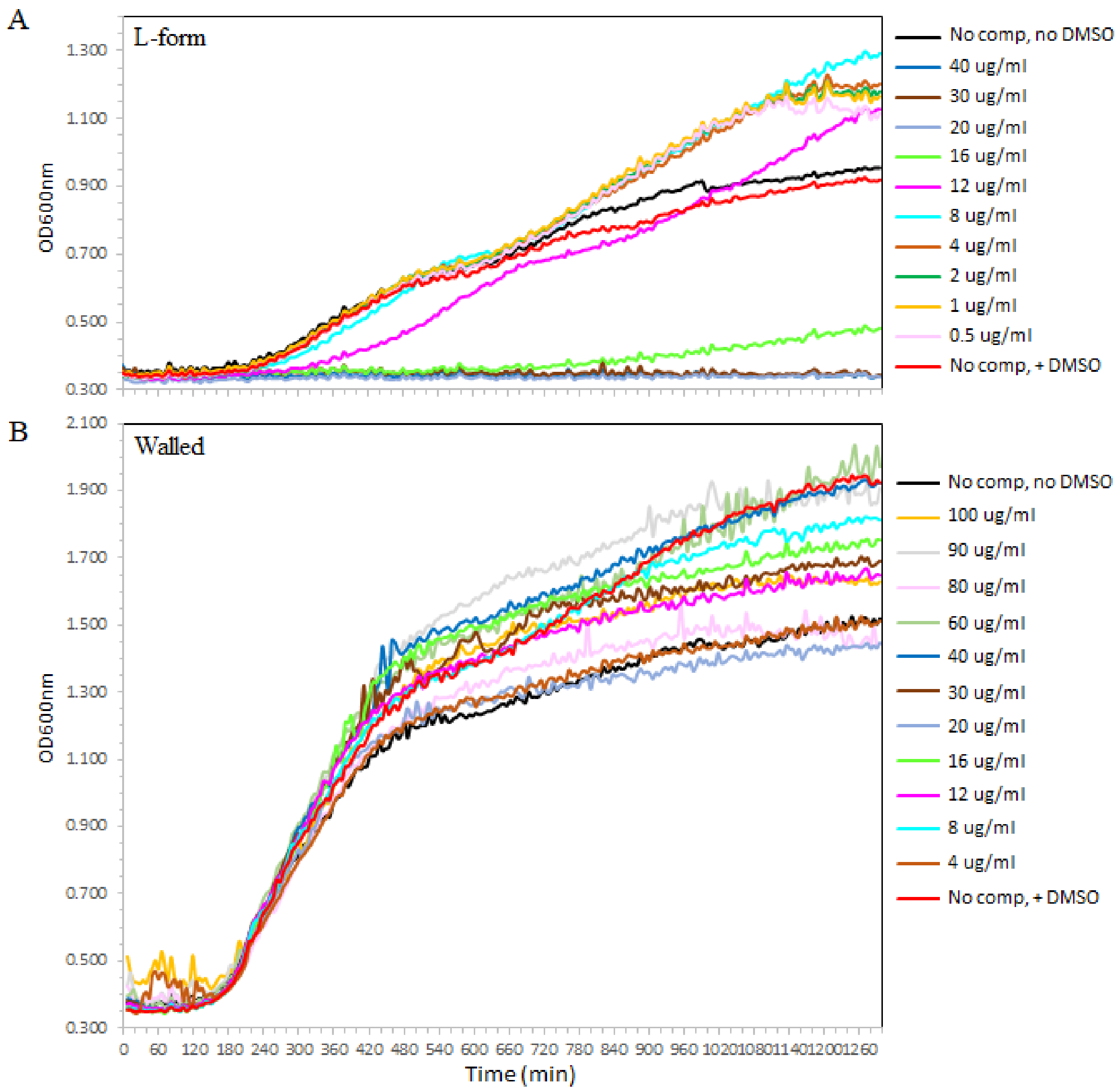

Figure S17. Growth curves of the L-form (top) and walled (bottom) *B. subtilis* in the presence of different concentration of demurilactone A. Experiments were done in 96-well microtiter plates.

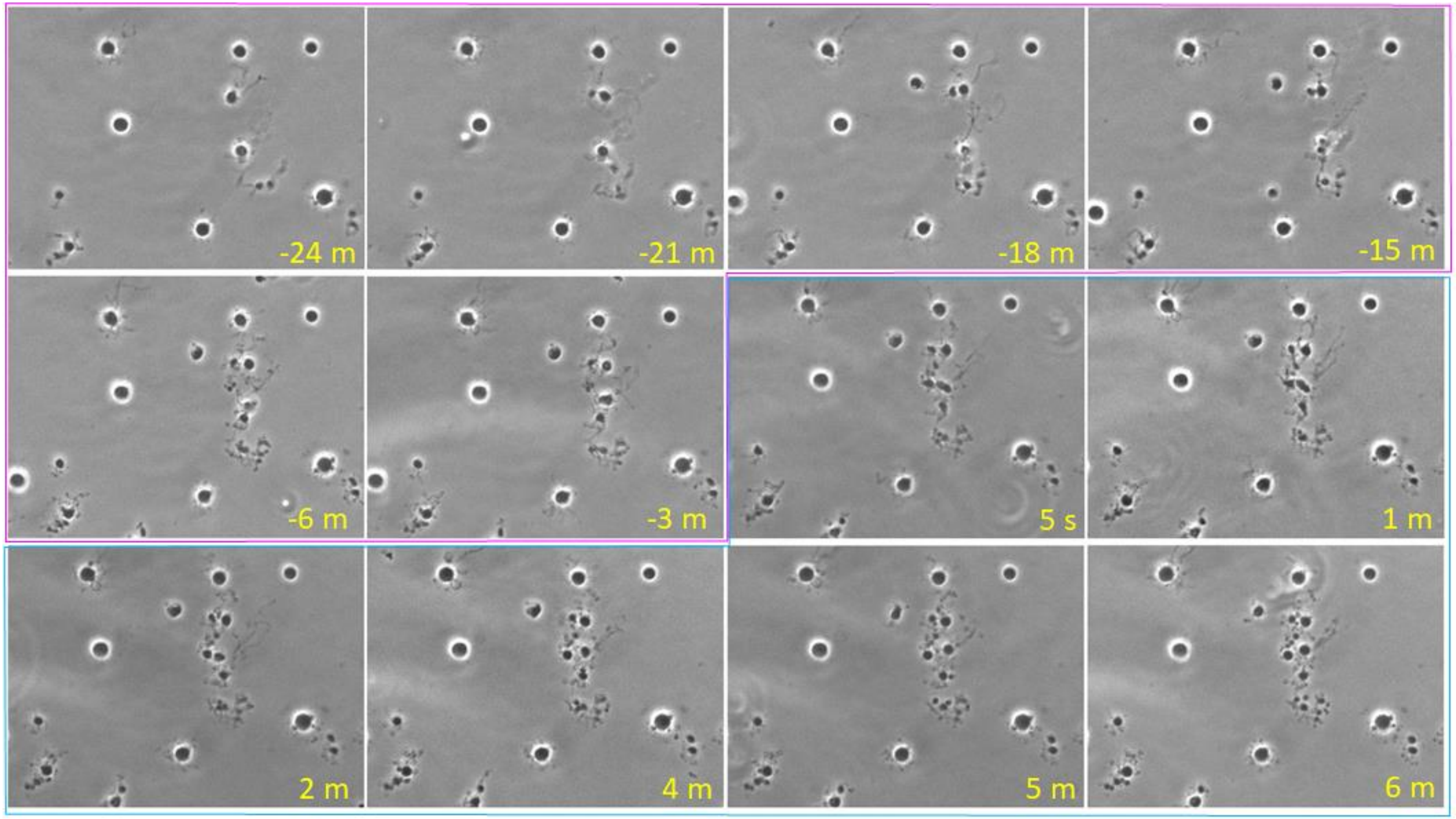

Figure S18. Time-lapse images of L-form *B. subtilis* before (framed purple) and soon after (framed cyan) adding demurilactone A (32 µg/ml). Times of imaging before and after the addition of the compound are labelled in yellow.
